# KUALA: a machine learning-driven framework for kinase inhibitors repositioning

**DOI:** 10.1038/s41598-022-22324-8

**Published:** 2022-10-25

**Authors:** Giada De Simone, Davide Stefano Sardina, Maria Rita Gulotta, Ugo Perricone

**Affiliations:** Molecular Informatics Group, Fondazione Ri.MED, Via Filippo Marini 14, 90128 Palermo, Italy

**Keywords:** Cheminformatics, Target identification

## Abstract

The family of protein kinases comprises more than 500 genes involved in numerous functions. Hence, their physiological dysfunction has paved the way toward drug discovery for cancer, cardiovascular, and inflammatory diseases. As a matter of fact, Kinase binding sites high similarity has a double role. On the one hand it is a critical issue for selectivity, on the other hand, according to poly-pharmacology, a synergistic controlled effect on more than one target could be of great pharmacological interest. Another important aspect of binding similarity is the possibility of exploit it for repositioning of drugs on targets of the same family. In this study, we propose our approach called Kinase drUgs mAchine Learning frAmework (KUALA) to automatically identify kinase active ligands by using specific sets of molecular descriptors and provide a multi-target priority score and a repurposing threshold to suggest the best repurposable and non-repurposable molecules. The comprehensive list of all kinase-ligand pairs and their scores can be found at https://github.com/molinfrimed/multi-kinases.

## Introduction

The kinase protein family is one of the most studied in literature because of the key role to many crucial biological processes such as cell division, signaling, and growth. Therefore, physiological dysfunctions of the kinases’ activity have been associated with human diseases^1^. Given the importance of these proteins, it is not surprising that their biological role and the selectivity of their modulators are extensively studied^2^. Indeed, this protein family has entered several drug discovery campaigns to treat cancer, cardiovascular, and inflammatory diseases.

Selectivity is carefully supervised when designing new drugs, in order to minimize adverse effects on off-targets and consequently to reduce the compound potential toxicity^3^. However, due to the high similarity of kinase binding sites, the design of novel selective inhibitors for a specific target still remains a challenge today^4^. A low selectivity may influence the clinical trials' progress due to high off-target toxicity. An example was the effort of dinaciclib, a CDK inhibitor, in the attempt to reach phase III clinical trial^5^.

Although the selectivity of a drug towards a specific target should be strongly considered in order to achieve the right balance between the success rate and the possible toxicity on the organism, on the other hand, a multitarget effect might have some interesting applications. In fact, recent polypharmacology studies suggest that the efficacy of a drug can be improved by specifically modulating multiple targets. In other words, a drug that "hits" several targets belonging to one or more pathways (network of interacting proteins) in some cases may represent a more effective therapeutic approach, by limiting the drawbacks generally deriving from the use of a single-target drug or from a combination of several drugs^6^. Indeed, a certain rate of drug promiscuity is even sought to repurpose drugs to new therapeutical targets. As a matter of fact, the binding site similarity between proteins is a crucial aspect to address in the repurposing process of known drugs. However, the right balance between protein binding site similarity and the number of targets that a known ligand can bind should be seriously considered in drug repositioning studies^7^.

In this context, the large amount of data available on public databases like ChEMBL^8^ and KLIFS^9^ has allowed the scientific community to direct efforts towards the use of computational methodologies, such as machine learning (ML) models, to improve the repositioning capabilities of known ligands. In fact, in the literature several ML and artificial intelligence (AI) approaches for bioactive ligands identification have been reviewed for protein families, such as G protein-coupled receptors^10^, and human diseases, like the Coronavirus Disease 2019 (COVID-19)^11^ and Alzheimer disease^12^. Furthermore, training of ML algorithms has also been reported to develop accurate models for epigenetic targets based on different fingerprint representations of compounds^13^.

In the last years, the application of AI in drug design and drug discovery has been largely adopted in order to exploit the big amount of data available to create affordable and reliable models. The effort in terms of cost and time in designing a clinical trial could potentially be reduced owing to the employment of AI techniques^14^. In a very recent work^15^, Paul et al. described the use of ML as a technology able to learn from human knowledge about a specific research area, and transfer patterns of information to an automated system, that can be effectively used in decision making alone or in conjunction with human expertise. An interesting trend was elucidated by Hay et al. in 2014^16^, where the success rate of drugs entered clinical development in phase I and subsequently approved by the US Food and Drug Administration (FDA) is nearly one-in-six. In the last decades, ML methods have also been used to address a wide variety of issues associated with the kinase protein family, such as predicting inhibitor activity profiles for a specific kinase^17^ or predicting different binding modes based on conformational kinase data^18^. In 2005, Briem et al.^19^ used an in-house dataset of compounds to predict the inhibitory activity on kinases without considering their selectivity. Recently, in the literature several works have been reported applying artificial intelligence approaches to classify and discover new kinases inhibitors starting from structure- and ligand-based approaches. Indeed, in 2020 Miljković et al.^18^ applied different machine learning approaches to generate models on the basis of compounds with binding modes confirmed by X-ray crystallography for predicting different classes of kinase inhibitors (including types I, I1/2, and II as well as allosteric inhibitors). Similarly, in 2021 Abdelbaky et al.^20^ described the application of predictive models to discriminate between four binding modes: three allosteric inhibitor modes (I, II, I1/2) and one non-allosteric mode. The high accuracy rate of both works demonstrated that the new machine learning models have considerable potential for practical applications. Furthermore, in recent years deep learning applications based on molecular fingerprints have also gained attention to support the virtual screening on specific protein families, such as kinases. An example is the EMBER method^21^, a novel molecular embedding made by seven molecular fingerprints to describe the same molecule. This approach employs a deep convolutional architecture that assesses ligands’ bioactivity on a data set containing twenty protein kinases with similar binding sites to CDK1. Jannsen and collaborators, in 2019 produced a great work based on a machine learning model approach to map the activity profile of compounds over the kinase family. Such an approach produced Drug Discovery Maps (DDM) created on the t-distributed stochastic neighbor embedding (t-SNE) algorithm. The latter was useful to visualize molecular and biological target similarity. This method was useful to explore target and chemical space and predicts the activities of novel kinase inhibitors^22^. In a recent work, Zhavoronkov et al.^23^ adopted a generative tensorial reinforcement learning (GENTRL), for de novo small-molecule design that revealed to be useful to discover new DDR1 kinase inhibitors.

However, to our knowledge, a multi-classifier based on the use of molecular descriptors to reposition ligands on the kinase family, appears to be lacking in the literature. In our opinion, such an approach could be useful in supporting results from classical computational methods, e.g., docking and pharmacophore approaches, to strengthen the activity prediction capability of the computational models. Moreover, a well-trained machine learning model could help in considering selectivity issues on the one hand, and repurposing on the other hand.

In this work, we present an extensive computational workflow tuned on available human kinome data to assess the predictive power of ML algorithms in classifying family-based active and inactive ligands and to evaluate its application for the repositioning of known kinase inhibitors.

**Figure 1:**
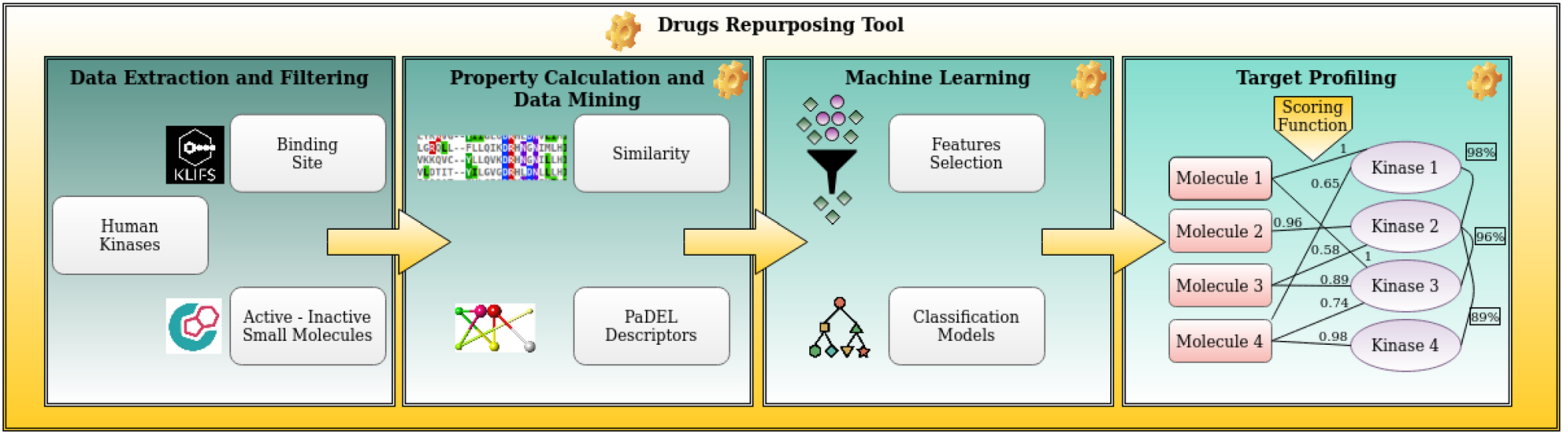
Workflow description of KUALA approach. The entire workflow is composed of four principal steps: (1) data extraction and filtering; (2) property calculation and data mining; (3) machine learning; and (4) target profiling with scoring function estimation for each predicted kinase-ligand pairs.

The workflow uses experimental kinase inhibitor data from the ChEMBL database^8^ and annotated by target and biological activity. The molecular descriptors have been calculated for each ligand using PaDEL^24^. This dataset, after careful data mining and curation step, has been used to train 12 well-known different ML methods in order to suggest a methodology to build the best classifier model with the best molecular descriptors set for each kinase. This study enlightened the importance of some specific descriptors useful for the classification of active kinase inhibitors and the best suitable classification model to be applied in this context. The combination of a multi-classifier methodology with a binding site similarity analysis into a scoring function has shown to be a valuable tool to further investigate the most promising predicted kinase-ligand pairs. Indeed, based on our studies the obtained results will be exploited for further analysis by entering a repositioning process. The entire workflow herein described is called Kinase drUgs mAchine Learning frAmework (KUALA) (see Fig. 1).

## Methods

### Data extraction and filtering

In this work, several databases have been employed and processed for the research activities described below. In detail, Kinase–Ligand Interaction Fingerprints and Structure (KLIFS) is a database which contains several information about kinase protein family, e.g., sequence alignment, structural kinase-ligand interaction, binding pocket and molecular fingerprints, and crucial residues within kinase-inhibitor selectivity patterns^9^. In this work, kinase structures including validated binding sites and their Uniprot codes were retrieved from KLIFS database. The overall number of human kinases within the database was 555. Proteins for which no binding pocket sequence information was known were filtered and removed. Each UniProt code was translated into the corresponding ID from ChEMBL database by using “Retrieve/ID mapping Tool” in Uniprot database^25^. ChEMBL collects large-scale information about bioactive molecules with drug-like properties^8^, and also includes the annotation of assays and targets.

Each kinase was annotated with unique ChEMBL ID, in order to retrieve ligand-related half maximal inhibitory concentration (IC_50_) activity values. All the activity data related to the collected kinases were experimentally obtained from different techniques and assays, which provided distinct but comparable concentration for each ligand–protein pair. In order to evaluate the activity, the minimum IC_50_ value was considered. Finally, unique pairs of kinases-small molecule were collected and molecules with IC_50_ below 0.03 µM were marked as active^26^, while those above 10 µM as inactive. The threshold chosen was the best trade-off between separation of active/inactive classes and loss of inactive ligand information. In addition, ChEMBL provides an "assay confidence score", which reflects both the type of target assigned to a particular assay and the certainty that the assigned target is the correct target for that experiment. For our analyses, only assay data with an "assay confidence score" of 9 were evaluated, that is those with a high degree of confidence assigned.

Both active and inactive small molecules (SMs) were filtered with Schrödinger nodes^27^ using the Knime^28^ platform (version 4.3.0). In particular, ligands with a number of carbon atoms higher than 7, with at least one aromatic ring and, finally, with a molecular weight in the range 100 to 700 g/mol were considered. To extract and process molecular information, it was decided to calculate molecular descriptors and not to use fingerprints, as they are not easily explainable. For this purpose, PaDEL-Descriptor (PaD) software^24^ was used to calculate molecular descriptors for each unique molecule (UM). The dataset consisting of ChEMBL IDs and PaD descriptors was analyzed for missing values number, variance, and zero elements number, thus filtering not available data and removing some descriptors. For each remaining descriptor, few missing values (less than 0.008% on complete ligands dataset) were replaced with the mean value. On the other hand, some descriptors showed infinite values for certain ligands, thus they were replaced with maximum descriptor value. Finally, the descriptors characterized by all zero entries were removed. In order to make an early features selection, Pearson’s correlation coefficient was computed for each pair of descriptors, then those strongly correlated with similarity above 0.9 were excluded^29^.

From a preliminary analysis, it was clear that the ligand dataset per single kinase was numerically unbalanced. Then, in order to achieve an adequate number of active and inactive compounds for each kinase, the proteins were filtered by considering a minimum number of ligands equal to 30. This number corresponds to a value between the 50th and 75th percentile of the distribution of the entire ligand set for all proteins, thus obtaining the final dataset consisting of 84 unique kinases (UKs) (Supplementary Data 1) with the related active and inactive compounds. The whole preprocessing and data extraction is depicted in Supplementary Fig. S1.

### Fingerprint calculation

In addition to the molecular descriptors, different types of fingerprints were calculated for each small molecule. In particular, the Molecular ACCess System (MACCS)^30^, Extended-Connectivity Fingerprints^31^ with radius 4 (ECFP4), calculated in the package RDKit, and Klekota-Roth Fingerprints in PaDEL-Descriptor software (KRFP)^32^.

MACCS are 166-bit long structural keys, which encode the molecule structure into a string of binary bits. Each bit corresponds to a structural characteristic, e.g., substructure or fragment in a predefined library. If the molecule has a predefined characteristic, the position of the bit corresponding to this characteristic is set to 1 (ON), 0 (OFF), otherwise. It is noteworthy that only the predefined structural keys in the fragment library are encoded.

An alternative to structural keys is hashed fingerprints such as ECFP4 (we used the 1024-bit long). Unlike structural keys, hashed fingerprints do not require a predefined fragment library. Instead, they are generated by enumerating through the molecule all the possible fragments that do not exceed a certain radius and then converting them into numerical values using a "hash" function. These functions are used to map data of arbitrary size to "fixed size" values. Therefore, enumerating all possible fragments in a molecule inevitably results in a "bit collision" that may lead to a loss of information, in which different fragments are converted to the same numerical value and to the same bit position. For this reason, there is no one-to-one correspondence between fingerprint fragments and bits, contrary to structural keys^31^.

KRFP are 4860-bit long corresponding to unique substructures predisposed to bioactivity^32^. Each bit encodes the presence or absence of a particular substructure (coded in SMARTS notation) within a molecule. KRFP can be used to identify the most frequent chemical motifs related to activity profile suggesting useful rules for molecules design.

### Feature selection

Molecular descriptors were used as feature variables in ML approach. In order to reduce the computational cost of training and improve the models' performance, several feature selection techniques were used. In particular, variable importance analysis with linear regression^33^ , Boruta^34^ algorithm and MRMR^35^, was performed.

### Variable importance analysis with linear Regression

The dataset was divided into subsets for each unique kinase by considering the related active and inactive ligands. Afterwards, the relationship between every selected feature and the ligand’s class were evaluated through generalized linear regression models with binomial error distribution and logit link function. Only statistically significant features with *p* values below 0.05 were maintained and called this set Linear Regression features (LRs).

### Variable importance analysis with Boruta algorithm

Boruta^34^ is a feature selection approach based on Random Forest (RF) algorithm, which considers all statistically valid variables obtained from the comparison between classification performance with respect to random variables. The approach creates pseudo-copies of all features and constructs an extended dataset trying to remove their correlations with the dependent variable. Finally, Boruta runs an RF classifier on this dataset and computes the Z-scores from standard error. Then, the maximum Z-score attribute (MZSA) among pseudo-variables is evaluated and compared to every feature. Those variables performing better than MZSA were labelled as ‘confirmed’. Conversely, the attributes with lower performance than MZSA were considered ‘rejected’ and removed. Finally, for each unique kinase, important variables confirmed from Boruta analysis were labelled Boruta Algorithm features (BAs).

### Variable importance analysis with minimum redundancy maximum relevance

Minimum redundancy maximum relevance (MRMR) is a widely used algorithm that has been conceived and effectively applied to microarray datasets to minimize similarity and to discover genes relevant to the phenotype^35^. It implements filter type feature selection by choosing the top k characteristics with the intuition that mutual information (MI) provides a useful measure of dependence between variables. Hence, the authors proposed to take only very dissimilar ones (minimum redundancy) and at the same time those that have high similarity with classification variable (maximum relevance). The minimum redundancy is obtained by minimizing the sum of MI between each pair of features while the maximum relevance is calculated from the sum of MI between each feature with the classification variable. Finally, the feature set is more representative of the classes they belong to.

The authors in Ding et al. compared several values of k. For the sake of completeness, we performed feature selection with MRMR (https://github.com/smazzanti/mrmr) considering k equal to 10, 50, 100, 150, 200.

### Feature sets

In order to compare the performance between the models trained using the above-mentioned variables ensemble, we decided to take into account only the best performing sets of features in terms of metric and computational cost, i.e., LRs, BAs, the common features (Cs) between them and, finally, their union (Us) as shown in Supplementary Fig. S2a.

### Machine learning models

For each feature set and for each protein, the following twelve well-known ML classification methods, i.e., Naïve Bayes (NB)^36^, Logistic regression (LR)^11^, Support Vector Machine (SVM)^37^, Decision Tree (C.50)^38^, Random Forest (RF)^39^, Neural Network (NNet)^40^, eXtreme Gradient Boosting (XGBoost)^41^, K-Nearest Neighbour (K-NN)^38^, Classification and Regression Tree (CART)^42^, Least Absolute Shrinkage and Selection Operator (LASSO)^43^, Ridge Regression (RIDGE)^44^, Elastic net regression (ELNET)^39^, were evaluated in order to train kinase-specific models (Supplementary Fig. S2b).

**Figure 2:**
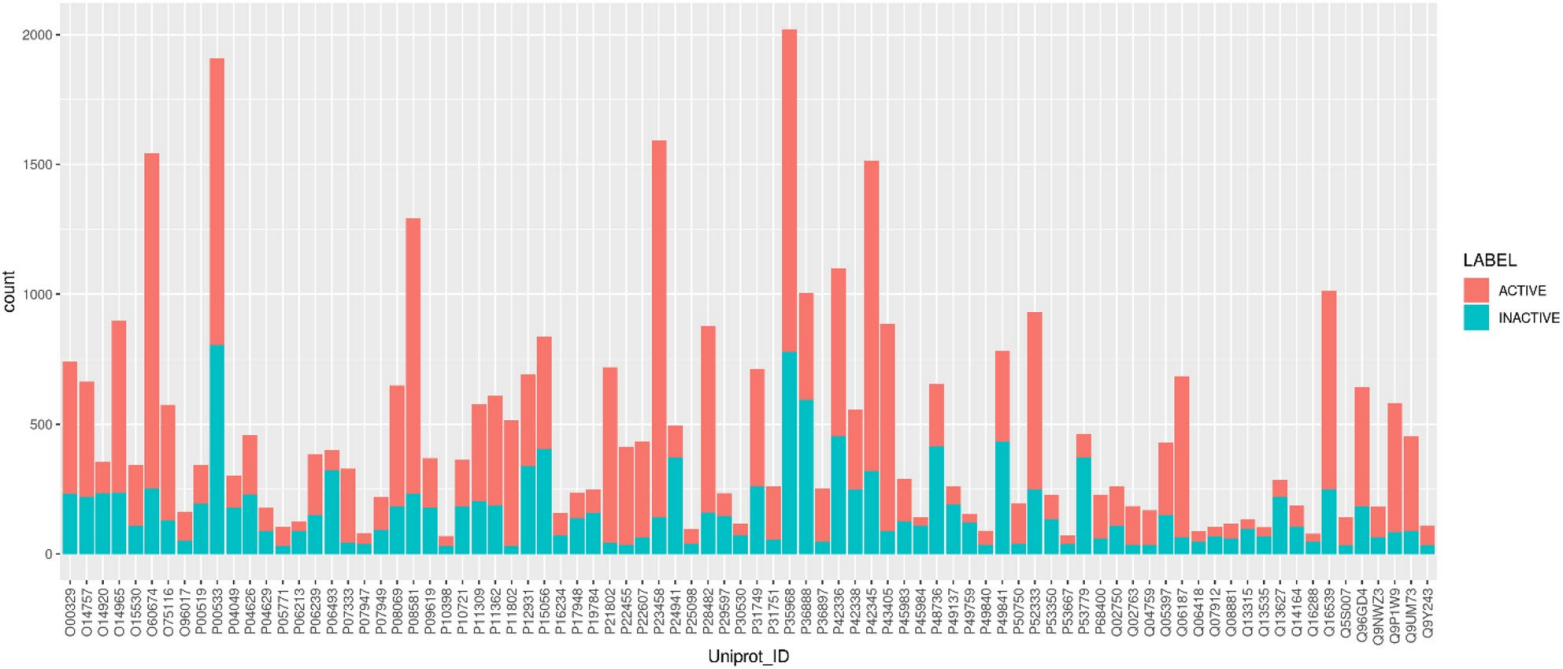
Number of active and inactive ligands for each protein of the 84 UKs. Extensive data analysis was carried out to balance the total number of active and inactive molecules. Some kinases, e.g., P35968, P00533 have a higher number of associated molecules.

Previously mentioned algorithms were performed in R (see full list of parameters in Supplementary Table S1). In order to take into account the inherent dataset imbalance (see Fig. 2), each method was accurately adjusted so that each class within kinase-specific training set, e.g., active, inactive, could be weighed appropriately. Also, several performance metrics were considered, i.e., Accuracy (Ac), Specificity (Sp), Balanced Accuracy (Ba), Precision (Pr), Recall (Re), F1-measure (F_1_) (Supplementary Fig. S3). The positive class was ‘active ligand for a kinase’, while the negative class was ‘inactive ligand for a kinase’. Therefore, true positives and true negatives represented the number of correct predictions of positive and negative classes, respectively. Similarly, false positives and false negatives counted the number of misleading predictions. All the above metrics were calculated for each kinase, classification model, and feature set. Finally, for every protein, the method that achieved the best performances and the related feature set were collected.

After the training and test phase of ML models, a further validation step was performed to validate the ML model capability to known active prediction. For this reason, a public database of kinase inhibitors currently in clinical trials (from phase I to IV), PKIDB^45^, and a screening library from MedChem Express vendor were downloaded. These datasets were filtered to remove the compounds used in the training phase. Moreover, only target belonging to the kinase set evaluated (84 UKs) were considered, thus obtaining an activity validation set consisting of 50 new single entries (drugs). Thus, these compounds were processed by the classifier to be labelled as actives or inactives.

### Ensemble model

In general, machine learning algorithms have their limitations and may be affected by high variance or low accuracy predictions. In order to reduce the error, without loss of generality, and to increase the performance of the learning model, it is often adopted an ensemble methodology. Such an approach may improve the predictive performance of a single algorithm by training multiple models and combining their predictions^46^. Based on these considerations, an ensemble learning was implemented starting from the above-mentioned ML models. For each feature set and for each kinase, all twelve ML classification methods were trained and tested in order to evaluate the performance and each prediction label has been collected. To estimate the decision process, an aggregation method that takes into consideration both performance and voting labels of all predictions was adopted. This approach computed a single aggregate label from the weighted average of models' performance trained with different feature sets grouped by each class label. Finally, the label which corresponds to the maximum vote was selected^46^.

### Multiple sequence alignment and kinase similarity assessment

In order to examine the kinases binding site similarity an alignment of all UKs binding pocket was carried out in Clustal Omega^47^ with mBed-like clustering guide-tree enabled. The results of these alignments were then considered for UKs similarity and identity computation with Sequence Manipulation Suite^48^. For similarity analysis, the following groups of amino acids with similar properties were considered, i.e., GAVLI, FYW, CM, ST, KRH, DE, NQ, P.

### Multi-target priority score

A multi-target priority score (*MTPS*) was created to prioritize ligand affinity towards kinases of the final dataset (84 UKs). MTPS takes into account two parameters:

1. The similarity between all the target known in the literature for that ligand (*P*_*j*_) and the one predicted by the model (*P*_*i*_)
2. The prediction model performance (*m*_*i*_)

For a given *l* ligand predicted as active on a generic protein *P*_*i*_, this score was calculated by using the following formula:

$$ MTPS_{i,l} = \frac{{\mathop \sum \nolimits_{j = 1}^{{N_{{P_{l} }} }} s_{i,j} }}{{N_{{P_{l} }} }}m_{i} $$

where *P*_*j*_ refers to the original protein target for *l* ligand, *s*_*i,j*_ is the similarity value between *P*_*i*_ and *P*_*j,*_ whereas *N*_*Pl*_ is the number of all the known protein targets for the *l* ligand. Finally, *m*_*i*_ is the performance metric value (e.g., F1-measure, Accuracy, etc.) associated with the goodness of the kinase-specific model for protein *P*_*i*_.

### Statistical analysis

A statistical-based approach was performed on the MTPS values in order to explore their distribution. In particular, the One-sample Kolmogorov–Smirnov test^49^ was used to assess normality in the overall multi-target priority score distribution. The null hypothesis of this test assumes no difference between the observed and theoretical distribution. A *p* value lower than 0.05 was considered to reject the null hypothesis.

Ameijeiras-Alonso excess mass test from package ‘modetest’ was performed in R to test the statistical significance of number of modes in MTPS distribution as well as to provide the estimation of the location of Modes and Antimodes and their density value. Modes represent the most frequent values in a dataset while the least frequent values between the modes are known as Antimodes^50^.

### Repurposing threshold evaluation

Statistical analysis results provided a tool to define a threshold in order to identify potential ligands for repositioning. Once evaluated the MTPS bimodal distribution and the significance of statistical tests, the second Mode value was selected as repurposing threshold (RT). In other words, ligands with an MTPS score higher than RT were flagged as a ‘reliable’ repurposing choice for a specific kinase.

### Docking screening of the predicted compounds

In order to perform docking screening, nine UKs were selected, i.e. proteins with UniProt IDs P31749, P25098, P42336, P06493, P08069, P28482, P07333, P21802 and P52333. For this purpose, the following PDB structures were downloaded according to the best resolution from the Protein Data Bank^51^ for each protein, respectively: 4GV1, 3V5W, 6PYS, 6GU2, 3O23, 1TVO, 6T2W, 6LVK and 3LXL.

Each PDB complex was prepared and optimized through the “Protein Preparation Workflow”^52^ of Schrödinger suite (Schrödinger, LLC, New York, NY, 2021, release 2021-3). The bond orders were assigned to the entire structure and the Chemical Component Dictionary was used when assigning bond orders to known het groups. Hydrogens were added to the structure. Bonds to metals were broken, zero-order bonds between metals and nearby atoms were added, and formal changes to metals and neighboring atoms were corrected. Disulfide bonds were generated and water molecules beyond 5 Å from het groups were deleted. Epik^53^ was used to generate het states at pH 7.4 ± 0.2 and, finally, H-bonds were optimized by using PROPKA^54^ at pH 7.4.

The predicted compounds for the nine above-mentioned UKs were prepared through LigPrep tool (LigPrep, Schrödinger, LLC, New York, NY, 2021) of Schrödinger suite. The employed force field was OPLS4^55^, the ionization states were generated at pH 7.4 ± 0.2 by using Epik. The molecules were desalted and tautomers were generated.

The docking grids of the nine selected proteins were created whereas each grid was centred on the ligand and the van der Waals (vdW) radii scaling factor of receptor atoms was set 1.0 with partial charge cutoff 0.25. Finally, these grids and the prepared ligands were used to perform docking screening by using the tool of Schrödinger suite. For this purpose, the selected protocol was standard precision and the employed ligand sampling method was flexible. The vdW radii scaling factor for ligand non-polar atoms was set 0.8 with partial charge cutoff 0.15.

## Results

### Data collection outcomes

After a first dataset physico-chemical preprocessing, 79,350 SMs were obtained and 422 out of the total 555 human kinases were collected and used for further analyses. These molecules were submitted to PaDEL software^24^ that returned a total of 1444 molecular descriptors (MDs) for each compound. Only the 1D and 2D molecular descriptors were calculated and the 3D ones were excluded from this analysis. This choice was adopted to avoid bias related to possible incorrect 3D ligand's conformers generated in silico. Indeed, a wrong 3D conformation could in fact affect the model training and the following prediction capability.

Among the 1444 total molecular descriptors, 195 MDs were characterized by all zero entries, so they were removed. Based on a correlation analysis, highly similar SM descriptors with Pearson’s coefficient above 0.9 were excluded, providing a final set of 532 descriptors.

Then, for each kinase-small molecule pair, the ligands were filtered based on IC_50_ values retrieved from ChEMBL database^8^, where compounds with IC_50_ ≤ 0.03 µM were marked as active^26^, while molecules with IC_50_ ≥ 10 µM as inactive^56–58^. These IC_50_ thresholds for active/inactive compounds provided a total of 48,928 unique kinase-small molecule pairs. Conversely, the final number of unique SMs was 33,503 and obtained a set of 378 unique kinases.

This analysis highlighted that the ligand dataset per single kinase was numerically unbalanced, as shown in Fig. 2. However, the dataset was not over-sampled to avoid overfitting. Thus, in order to achieve an adequate number of active and inactive compounds for each kinase, the proteins were filtered by considering a minimum number of ligands equal to 30 as threshold, i.e., at least 30 active and inactive SMs, corresponding to a threshold between the 50th and 75th percentile of the entire protein set. This data curation provided overall 40,483 unique pairs (kinase–ligand–label: active or inactive) while the final number of Unique Molecules (UMs) was 29,792. Finally, the dataset for our study was composed of a total of 84 UKs. Figure 2 illustrates the number of active and inactive ligands for each protein of the 84 unique kinases. As observable, it is evident that the dataset with annotated thresholds of 0.03 to 10 µM was still unbalanced in terms of the number of active and inactive ligands. We wondered if a threshold of 25 or 50 µM for inactive molecules might generate a balanced ligand distribution and consequently improve the model training. However, we were able to prove that the trouble of the dataset imbalance still remained, since it should be ascribed to the available kinase data. Therefore, it was preferred to proceed with the choice of 0.03 to 10 µM as IC_50_ range for active compounds in an attempt to populate ‘actives’ and ‘inactives’ classes as much as possible.

### Variable importance analysis

The ligand dataset generated from the above-described data curation was further processed by performing a variable importance analysis. For this purpose, the importance of each molecular descriptor was calculated with reference to the protein of interest, both by fitting generalized linear models and by comparing the importance of the original attributes against the random variable, estimated using their permuted copies (Supplementary Fig. S4). Statistically significant and confirmed features were selected for further analyses.

Moreover, Supplementary Fig. S5 depicts the distribution of sizes of the different feature sets (Boruta, Linear Regression, Common and Union) for each kinase. As observable, the number of molecular descriptors related to Linear Regression algorithm is higher compared to Boruta feature set. This fact is likely due to the substantial difference between the two methods applied for the feature selection process. Indeed, the Boruta algorithm is in general more restrictive because it employs a Random Forest-based approach and creates pseudo-copies of all features, in order to remove uninformative variables.

MRMR was not included in the analysis since it was not computationally feasible to compute all feature set for each k value by considering the size of our dataset. Notably, the features set selected starting from a given value of k includes the same features already selected for values less than k, i.e., the features set identified by setting k = 50 is a superset of that detected with k = 10 and so on.

### Machine learning performance

The kinase-specific ligand descriptors selected were used as features in order to train twelve ML models employed for this study, i.e., Naïve Bayes (NB)^36^, Logistic regression (LR)^11^, Support Vector Machine (SVM)^37^, Decision Tree (C.50)^38^, Random Forest (RF)^39^, Neural Network (NNet)^40^, eXtreme Gradient Boosting (XGBoost)^41^, K-Nearest Neighbour (K-NN)^38^, Classification and Regression Tree (CART)^42^, Least Absolute Shrinkage and Selection Operator (LASSO)^43^, Ridge Regression (RIDGE)^44^, Elastic net regression (ELNET)^39^. This training process was repeated for each feature set, namely BAs, LRs, Cs and Us. In order to evaluate ML models performance, each kinase-specific dataset was divided into training and test sets with a 70–30 split, such that the results were comparable and the number of active and inactive ligands was representative of the initial dataset. The hyperparameter tuning phase, where applicable, was model-dependent, since the best parameter values were chosen based on the results obtained from grid search algorithm. For example, for ‘kernel’ parameter in SVM, firstly, linear, polynomial and sigmoid kernel performances were compared with each other and sigmoid kernel was selected as the most appropriate. Finally, grid search for gamma, cost and epsilon values were carried out. Conversely, the performance of NNet was assessed by considering the number of hidden neurons and layers.

Once the models were appropriately tuned, the kinase-specific test set was used to validate the model and fill a confusion matrix in order to compute several evaluation metrics (see Machine learning models section for details). For a couple of kinases, it was not possible to train some models, therefore the performance in these cases was not evaluated. Finally, the performance results were collected and are depicted in Supplementary Fig. S6a. The acceptance of metrics is dependent both on the class balance of the original dataset and on the aim of the study. Thus, accuracy should guarantee good performance for balanced dataset, while recall might be useful when evaluating the ability of a model to find all the active ligands within a dataset. Precision is most suitable to identify the rate of success when predicting the activity of a ligand, on the other hand, specificity can be employed to assess the probability of a negative test.

**Figure 3:**
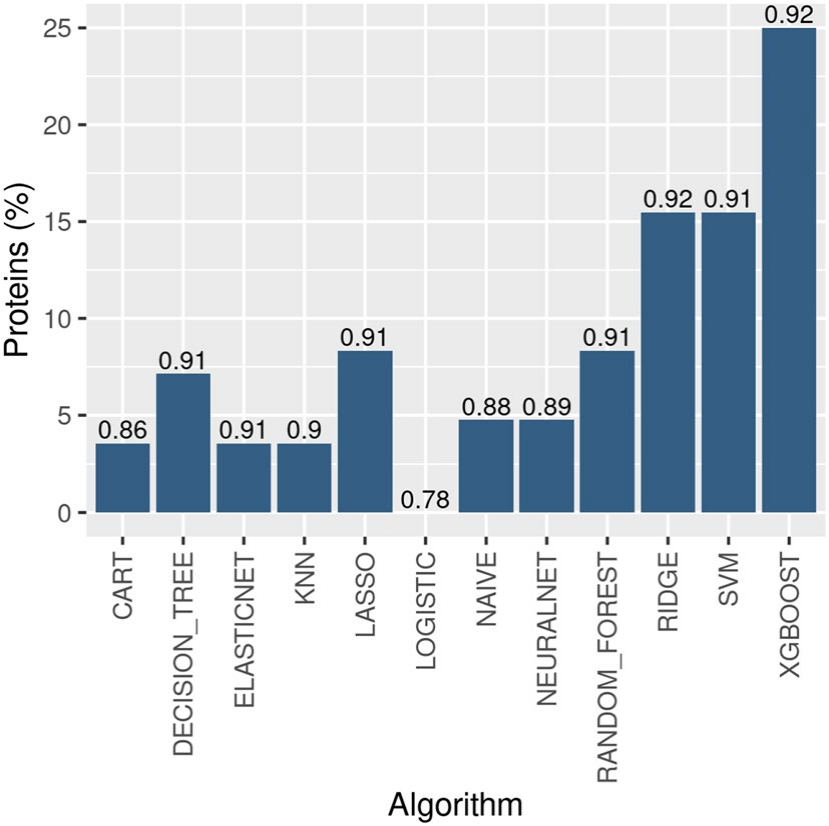
Best performing algorithms for F1-measure. For each algorithm, the Y-axis indicates the percentage of kinases for which the corresponding model in the X-axis achieved better performance compared to the others. Whereas the values shown at the top of the bars represent the overall average of F1 and each algorithm for all proteins in UKs.

In the light of the above, F1-measure was preferred among the other metrics due to its capability to consider the dataset imbalance^59^. With this in mind, the best methods (BMF1) for each kinase were defined as pair of ML algorithm-feature set that reported the highest F1-measure. Figure 3 illustrates the percentage of proteins within our 84 overall UKs, so that the given algorithm got the best results, in terms of F1-measure, with a given feature set (BAs, LRs, Cs, Us). From the analysis of the model performances, the higher average F1-measure was obtained by XGBoost and RIDGE algorithms, followed by SVM, even if only XGBoost had the highest average score. Specificity for all models had lower values on average (Supplementary Fig. S6a). This might be motivated by the fact that for many kinase-specific training sets the number of inactive ligands was lower than the active ones as depicted in Fig. 2.

The performance of models calculated through the use of fingerprints, e.g., MACCS, ECFP4, and KRFP are reported in Supplementary Fig. S6b, c, and d, respectively.

For an exhaustive analysis, the distribution of BMF1-related feature sets among the kinases is depicted in Supplementary Fig. S7. On average the number of kinases that selected BAs as best feature set was about three-fold lower than those preferring LRs. This was probably due to the substantial difference between the two algorithms. Furthermore, on average, the number of descriptors selected by the linear model was comparable with those annotated as ‘union’. This suggests that the descriptors selected by the Boruta algorithm might indicatively overlap to the LRs.

### Most frequent descriptors

**Figure 4:**
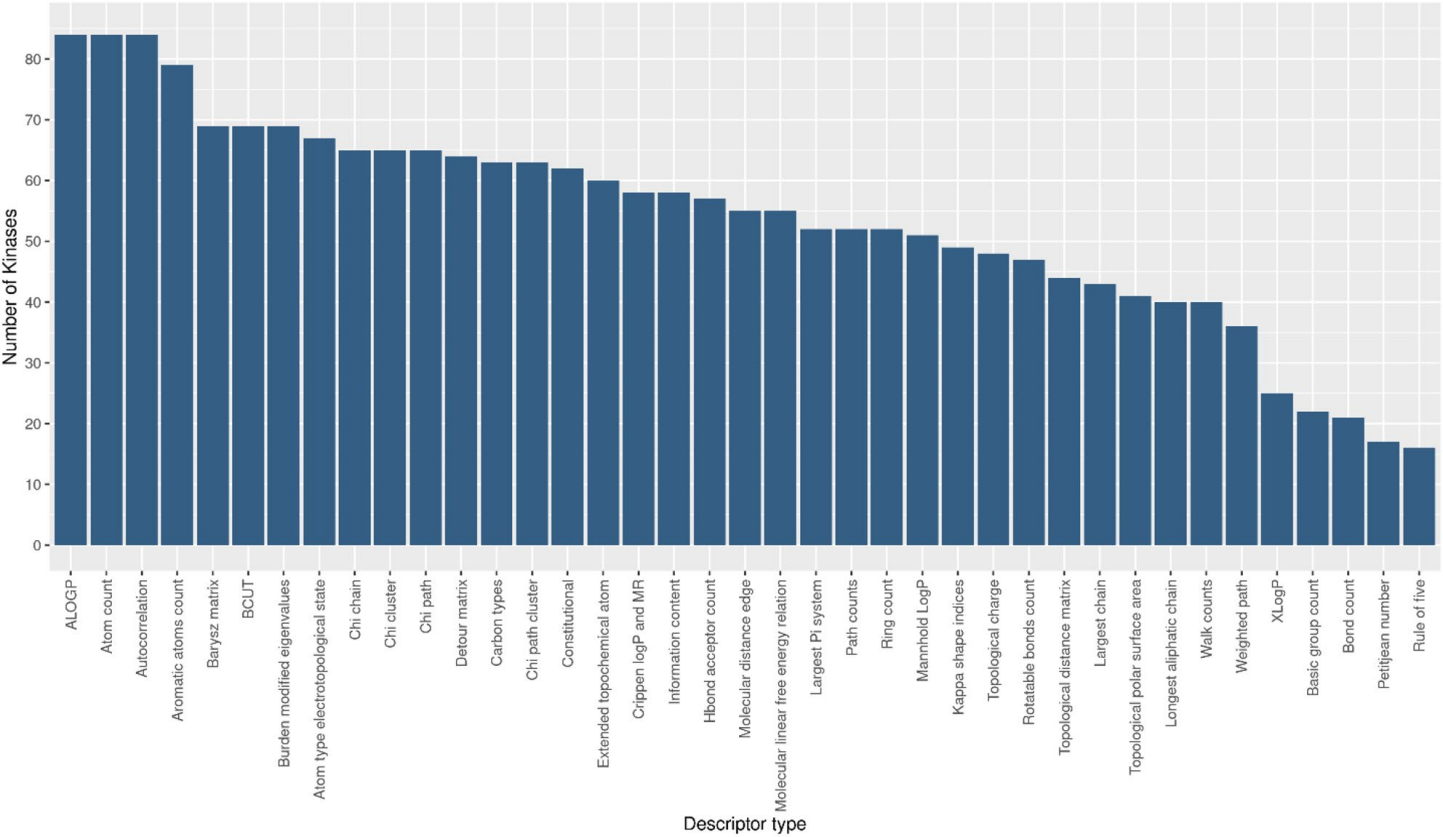
Most frequent descriptors type. The most frequent descriptors emerged from feature selection used to train the BMF1 models related to the 84UKs used in the study.

The above-described analyses shed light on a group of molecular descriptors that were evaluated as ‘best’ in terms of the F1-measure value for each protein. For an exhaustive analysis of the results, these descriptors were further examined to understand the possible kinase-ligand binding. For this purpose, we used a family classification provided by PaDEL^24^. In particular, PaD descriptors have been grouped by family with 39 different types of 2D descriptors. The basic idea was that for a given protein model the related actives share common chemical, topological, geometric, symmetrical or physical characteristics, which are then translated into numerical values of molecular descriptors. Therefore, we wondered if the descriptor families were all equally shared by our protein dataset or whether there were descriptor’s families specific only for some proteins. In Fig. 4, the descriptor classes, which most frequently emerged from feature selection, are shown. In detail, the class of descriptors ‘AlogP’, ‘Atom Count’, ‘Autocorrelation’, ‘Aromatic atoms count’, ‘Barysz matrix’, ‘BCUT’, ‘Burden matrix’, and ‘Atom type electrotopological state’ were present within each kinase-specific feature set. Moreover, this result was comparable with the outcome of Abdelbaky et al. work, where the authors found that these descriptors reported a good ability to differentiate between the active and inactive classes within the kinase family^20^. This would suggest that these descriptors could well characterize the inhibitors of the examined kinases.

In detail, ‘AlogP’ descriptors provide an estimation of atom-based hydrophobicity and hydrophobic interactions in protein − ligand complexes^60^. Indeed, known kinase inhibitors have been extensively shown to generate hydrophobic contacts with their targets^61^, by participating into the ‘hydrophobic spine’ network typical for the protein kinase core^62^. Furthermore, the ‘Autocorrelation’ descriptors are related to the spatial distribution of molecular properties, such as polarizabilities associated with highly electronegative elements present in a compound^63^. In Arthur et al. work these descriptor family has been positively associated with the bioactivity of the compounds, hence, by increasing the magnitude of these descriptors, the activity of the molecules is also increased^64^. Moreover, ‘Barysz matrix’ family accounts simultaneously for the presence of heteroatoms and multiple bonds in the molecule, while ‘Burden matrix’ descriptors consider atomic properties, such as electronegativity contributions, and bond orders (single, double, triple or aromatic bonds) for pairs of bonded atoms^65^. In this context, examples of the importance of these descriptor families for the kinase inhibitors have been reported by Ikwu et al.^66^ and Pourbasheer et al.^67^. Finally, the ‘Atom type electrotopological state’ encodes structural attributes, such as the topology and electronic environment of molecular fragments, correlated to various activity responses of the ligands^68^.

It should be also noted that among the overall 55 2D PaDEL-classified descriptor families, the model returned only 39 as the most frequent of the analyzed proteins, the remaining 15 did not report descriptors evaluated as relevant for any kinase model.

In the above analysis related to the molecular descriptors families, the features selected by Ensemble and fingerprint models have not been included since it is not possible to trace the structural characteristics.

In fact, each Ensemble model directly depends on several ML methods and feature sets while for what concerns the fingerprints, although it is always possible to calculate the frequency each bit is selected with, the information contained within each bit is not directly comparable with the explainability of molecular descriptors. Furthermore, as reported in literature, it is not, indeed, recommended in case of extended topological or chimico-physical exploration of molecules, i.e., rational molecular design^69^.

### Validation of ML model prediction capability

**Figure 5:**
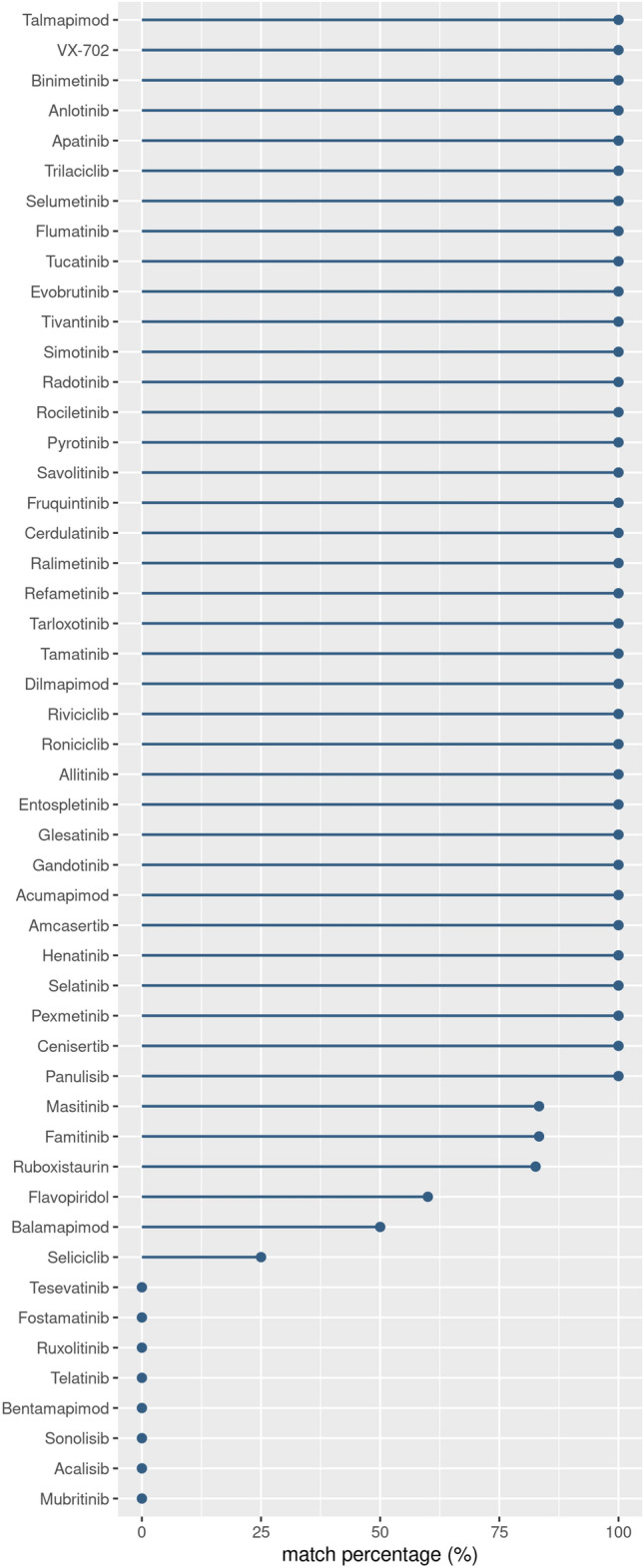
Validation results of the BMF1 on the known kinases inhibitors. PKIDB and MedChem Express datasets of kinase inhibitors were fed to the 84 BMF1 to validate their predictive potential. KUALA is able to correctly identify active inhibitors in 72% of cases.

Classification results were further assessed by using a public database of kinase inhibitors currently in clinical trials, from phase I to IV, PKIDB^45^, and a screening library from MedChem Express vendor. This dataset was filtered by neglecting compounds used in the training phase, and considering only targets belonging to 84 UKs. The resulting 50 new single entries (drugs) were experimentally active on one or more targets of our datasets. Thus, this validation dataset was exploited to test BMF1, where the framework was able to correctly assign the ligand as ‘active’ to each of the validated targets in 72% of cases (percentage of validated target matching 100%). In 12% of cases, the model was able to classify the ligand as "active" for a fraction of its validated targets (percentage of validated target matching between 50 and 83%), and finally, only in the remaining 16%, it failed to produce the desired results. Figure 5 shows the validation procedure results sorted by the highest number of kinases for which the models predicted the drug as active. As shown, the proposed methodology allowed us to obtain results that were compatible with the experimental data.

For the sake of completeness, the validation dataset was fed to the other methodologies, e.g., mRMR, Ensemble, ECFP4, MACCS, and KRFP. Then the classification results were compared with KUALA in terms of capability of computed models to correctly label the related inhibitors. Supplementary Fig.S8 shows a heatmap ordered by percentage of target match obtained by all the selected methodologies for each drug contained in the validation dataset. In general KUALA achieved the best results and for a few inhibitors they are comparable only with the Ensemble method. Conversely, models obtained from MACCS fingerprint didn’t provide any prediction for 4 drugs (Henatinib, Apatinib, Anlotinib, Telatinib) since they were not evaluable. For several inhibitors, e.g., Fruquintinib, Tivantinib, Apatinib, KUALA predicted all their targets contained in the 84 UKs while other methodologies achieved very low percentage of match. Moreover, KUALA is the only able to classify 50% of Balamapimod’s targets. Nevertheless, Ruxolitinib and Fostamatinib are correctly classified by all other methods except for KUALA. Overall, considering the global results and the percentage of match, KUALA for 36 inhibitors is able to predict 100% of targets, followed by Ensemble (35 inhibitors), ECFP4 (33 inhibitors), KRFP (33 inhibitors), mRMR (31 inhibitors), and finally, MACCS (29 inhibitors).

### Kinase similarity assessment and kinase-predicted ligand prioritization

An interesting aspect in drug repurposing is the binding site similarity between targets. Therefore, the similarity between pocket sequences can be used to identify new targets^70,71^.

In the light of above, we decided to build a multi-target priority score (MTPS), that includes a pairwise similarity between pocket residues of the 84 kinases, the number of known targets for each ligand and, finally, the classifier performance (i.e., F1-measure).

For each kinase, the dataset used for the MTPS calculation was tuned removing compounds considered during the training phase.

In synthesis, our framework was applied again on each protein to search for actives repositioning. These calculations provided a list of novel potential active compounds for each kinase. Finally, these predictions were fed into a ‘classification matrix’, that was subsequently used for the scoring function computation.

For each UK, the classification matrix was matched with the activity data from ChEMBL database^8^ to identify the known targets for every ligand.

Then, a binding site similarity was computed between the known targets of a current ligand and the protein that predicted this molecule as active. On average, the binding pockets shared the same amino acids by 34.37% (Supplementary Fig. S9). In Supplementary Fig. S10 the hierarchical clustering of pairwise UKs binding pockets similarity is shown where binding pockets had 52.40% of amino acid similarity.

Finally, using the similarity values, the MTPS scores were computed for each predicted protein–ligand pair. This scoring function allowed us to rank the putative active compounds for a specific kinase.

Therefore, when the MTPS score is high, it means that ligand identified by the BMF1 might be considered as a ‘reliable’ repurposing choice for that protein. Conversely, when the MTPS is low, it suggests that a certain ligand might be very promiscuous, thus it might not be optimal for repurposing because of the risk of toxicity related to off-targets.

In order to rationalize the results and discriminate optimal from non-optimal ligands to consider for repurposing, the MTPS scores were further examined. The statistical analysis demonstrated that MTPS values did not approximate normal distribution (Kolmogorov–Smirnov *p* value < 2.2e− 16). Conversely, Ameijeiras-Alonso excess mass test confirmed a bimodal distribution.

This trend is shown in Supplementary Fig. S11 where two modes are clearly visible centered at 0.34 and 0.54, respectively. The higher density value corresponds to the second mode which represents a good repurposing threshold (RT) separating ‘reliable’ from ‘unreliable’ ligands.

In this case RT, relying on a statistical test, allows to reduce the error, that is the proportion of ligands that more likely can be selected for repositioning.

Once a statistical methodology is established in order to define a threshold, we confidently filtered predicted active ligands for the kinases under study.

**Figure 6:**
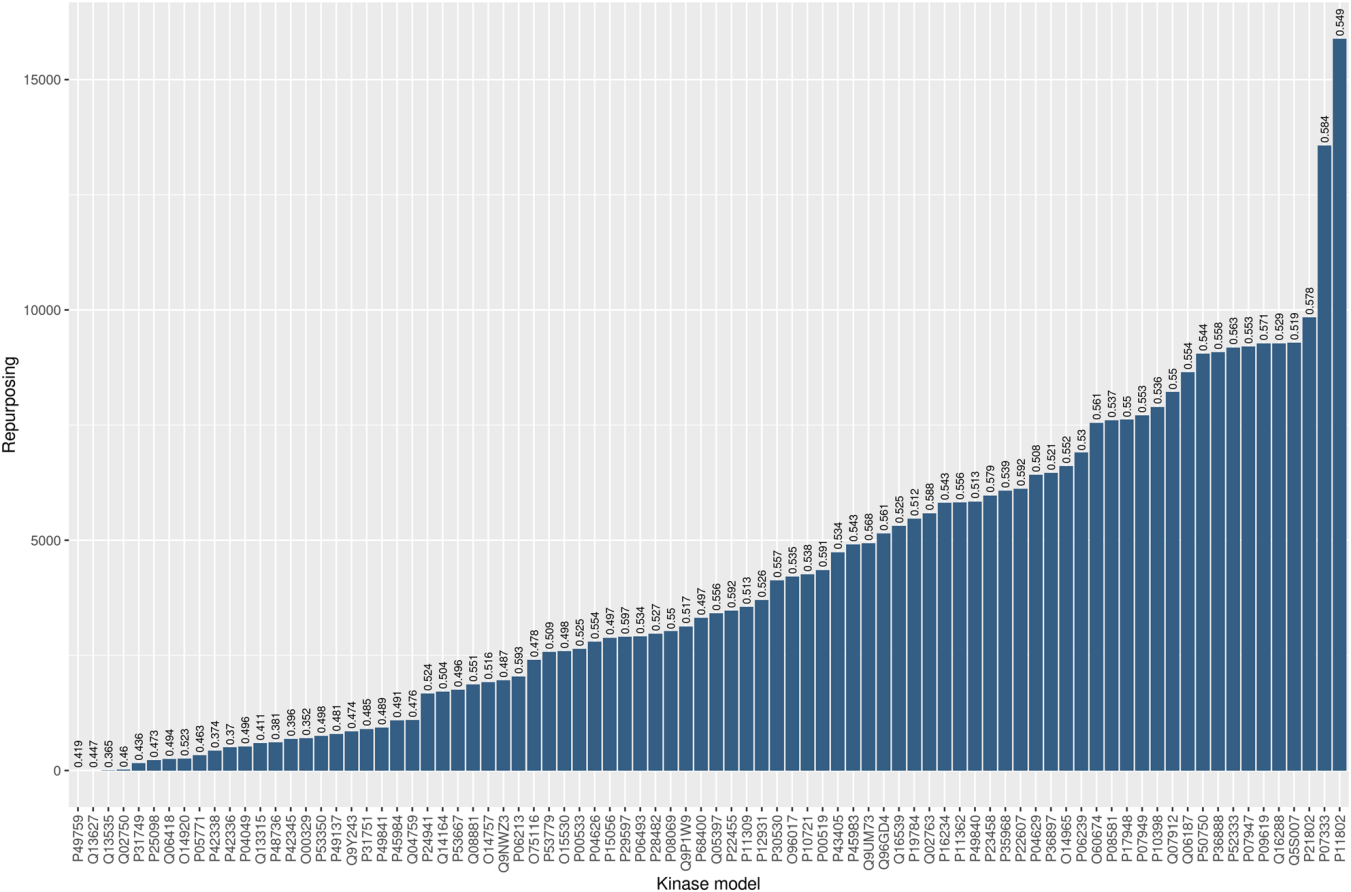
Predicted actives distribution related to 84 UKs models. In the X-axis the BMF1-related kinases are reported, while in Y-axis the number of repurposable ligands which achieved an MTPS score higher than repurposing threshold (RT) is reported in ascending order. On top of each bar the average MTPS score for each predicted protein–ligand pair is reported.

Figure 6 shows the ‘reliable’ ligands distribution for all 84 kinases where on top of each bar the mean multi-target priority score is reported. It can be seen that there is a wide range of variability among the proteins. For example, the BMF1 for P11802 (CDK4 protein) predicted a total of 29,275 compounds of which 15,883 were labeled as ‘reliable’ based on MTPS value. This high number of predicted actives depends on the high similarity of CDK4 with the other kinases within the dataset. Furthermore, it is also noteworthy that many of the predicted molecules on CDK4 are known actives for the most similar kinases retrieved in this workflow. This outcome for CDK4 is depicted in Supplementary Fig. S12, where the distribution of the actives is illustrated as a function of the most CDK4-similar proteins. Conversely, as discussed above, P31749 (AKT1 protein) exhibited an opposite trend and, consequently, a low number of potential repurposable ligands (Supplementary Fig. S13).

Therefore, the distribution in Fig. 6 provides crucial insights for drug repurposing and promiscuity. The rightmost bars suggest that the protein interaction sites (e.g., for P11802 and P07333) might be very promiscuous by binding a high number of UKs known actives and these proteins might not be suitable for entering a drug repurposing process. On the contrary, the leftmost bars refer to kinases reporting a low number of similar proteins, e.g., Q13535, Q02750 and P31749, and a very low number of predicted compounds that are also active for other kinases. In Table 1, as an example, we provide a comparison between some structures of predicted actives and known active ligands and/or drugs for some of the kinases on the left tail of Fig. 6.

**Table 1:**
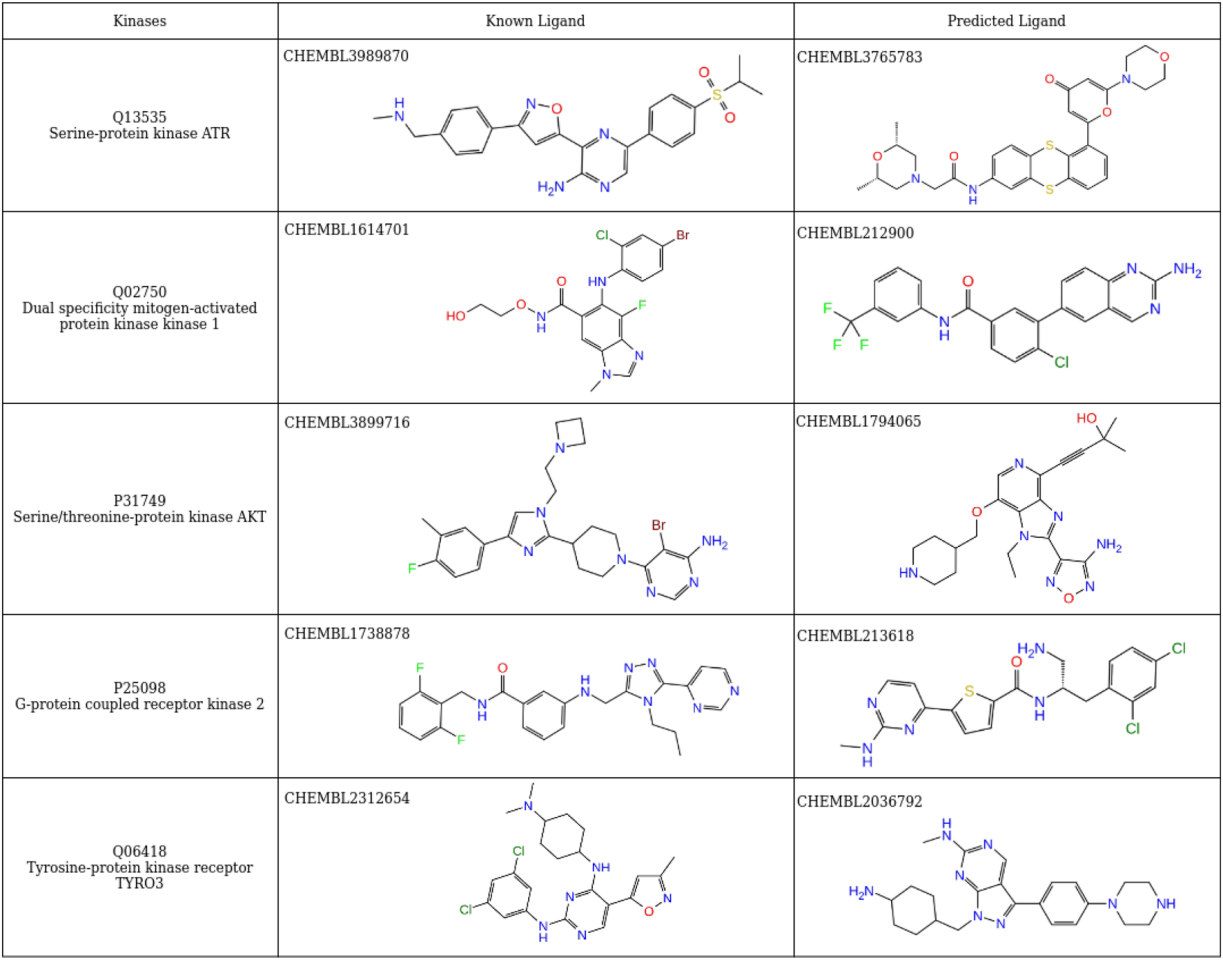
Predicted and known actives for kinases.

A comparison of the structures of predicted active ligands for kinases Q13535, Q02750, P31749, P25098, Q06418 and the structures of some of their known actives.

**Figure 7:**
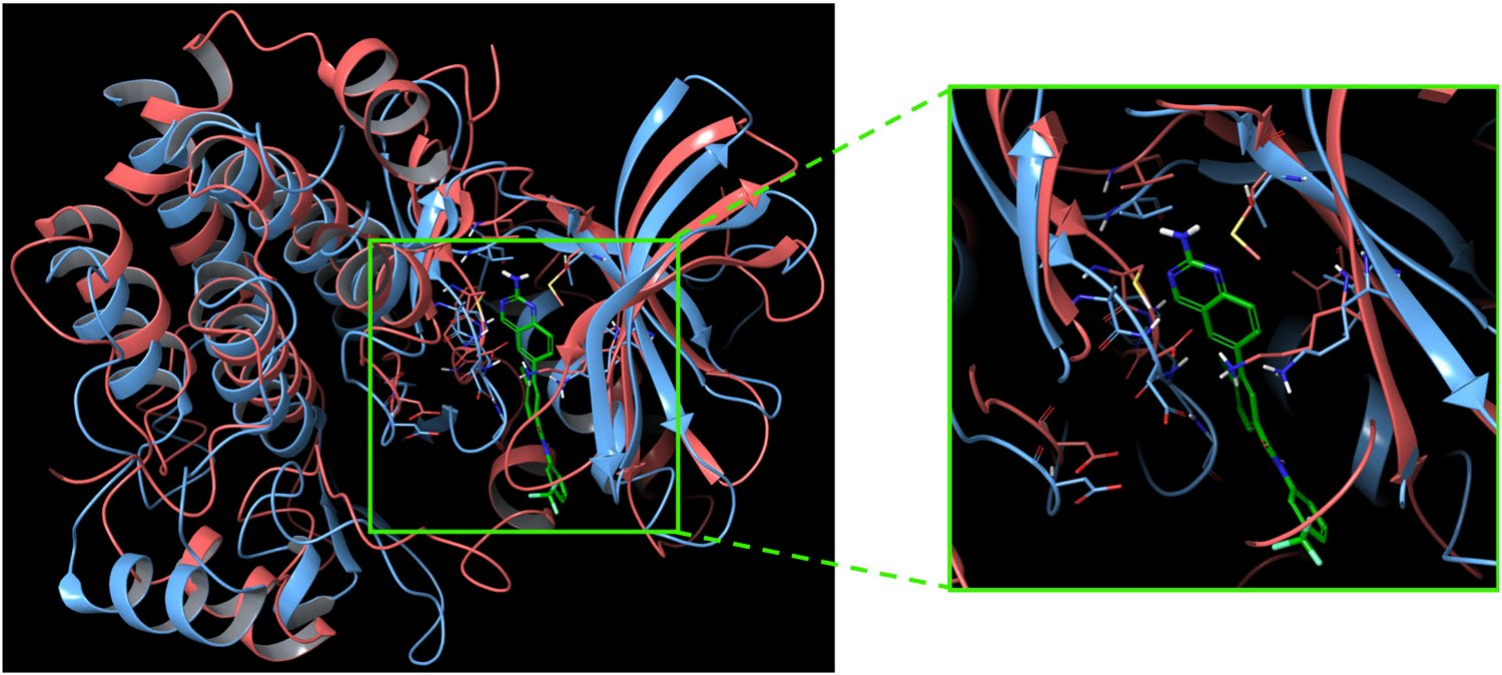
Superposition of pocket amino acids belonging to similar kinases. On the left, superposition of binding site amino acids from the original MAP2K1 target (UniProt ID: P06239, orange chain from PDB 1S9J), complexed with its ligand CHEMBL212900 (green molecule), and the potential novel target, LCK protein (UniProt ID: Q02750, cyan chain from PDB 1QPC); on the right, a close-up of the superimposed binding site residues.

Furthermore, Fig. 7 illustrates an example of similar binding sites (identity 35.29% and similarity 56.47%), where, for a given compound (CHEMBL212900), the pocket amino acids of the original target (MAP2K1 protein) have been superimposed to residues from a novel potential target (LCK protein). A complete list of all predicted compounds for each kinase accompanied by RT thresholds can be found https://github.com/molinfrimed/multi-kinases.

Finally, in order to further explore the reliability of the above-mentioned results, a group of proteins was selected from the distribution plot in Fig. 6 to perform docking screening of the predicted molecules. For this purpose, overall, nine UKs were chosen from the left tail (UniProt IDs P31749, P25098 and P42336), the center (UniProt IDs P06493, P08069 and P28482) and the right tail (UniProt IDs P07333, P21802 and P52333) of the plot according to the availability of the PDB structures including co-crystallized compounds within the binding sites. Most of the predicted compounds were able to reproduce all or the majority of the interactions observable from the PDB complexes. It is also noteworthy that for some of these kinases the top-ranked compounds according to MTPS values exhibited chemotype strictly similar to the known active molecule complexed within the PDB structure. Supplementary Table S2 depicts a comparison between the interaction diagram of co-crystallized ligand and an example of a top-ranked molecule through docking screening. The above-mentioned results highlight the importance of the ML and non-ML methods integration when running virtual screening for repurposing or for the molecular design in general. The ML approach, based on 2D molecular structure, helps in this case to find chemotypes compatible with pharmacophore moiety of the ligand, the structure-based analysis reinforces the prediction capability. Testing predictions in the 3D binding site environment. KUALA was actually designed as a first step tool to guide the rational and explainable molecule selection for subsequent drug design steps.

## Discussion

In the last decades, the issue of familywise bioactive ligand identification and characterization has gained increasing importance within the research community^10^, especially when referred to polypharmacology or drug repurposing issues^72,73^. In our computational workflow, data extraction from KLIFS and ChEMBL databases were used to explore structural and physico-chemical properties distinctive for kinase active ligands. Moreover, the methodology used led to the definition of kinase-specific feature set and ML models. Every ML method achieved slightly different results for each metric and no one clear ML algorithm-feature set pair is able to reach the best performance. Thus, among the ML employed approaches, XGBoost and RIDGE have been shown to correctly and better detect active ligands based on F1-measure, as previously shown in Fig. 3.

On these premises, ensemble learning has been taken into consideration. However, the use of this approach increases the computational time and especially excludes the possibility to trace the structural and topological properties of repurposed ligands for a specific kinase. Briefly, for 64% of kinases, Ensemble approach represents the best choice with an increase in performance of 3.01 ± 2.63%. This is a good result but is not sufficient to implement a methodology entirely based on such an ensemble method. Moreover, when used to classify known drugs it does not outperform KUALA.

In a multi target framework like KUALA, if on one hand it is always possible to add algorithms to make the methodology more robust and reliable, on the other hand it is equally important to maintain the link between the structural features of molecules and their classification as actives/inactives.

The feature selection represents an important step for the model's creation. Among the most used algorithms it was considered also MRMR^35^, that is based on minimum number of features that are maximally dissimilar to each other. This method is useful when it is necessary to specify a fixed number of features k. In our scenario, each kinase induces a specific feature set with different sizes in order to better build the model. Setting this parameter to a unique value for all proteins would lead to a very likely decrease in performance. Boruta provides a clear methodology to filter out the uninformative features while in MRMR is difficult to set k value a priori and evaluate all possible k is not feasible when dealing with multiple algorithms. In general, no single fixed k value is the best choice for all kinases. As k varies MRMR is able to provide improved results for small subset of proteins, e.g., k = 10 for 7.14%, k = 50 for 10.71%, k = 100 for 5.95%, k = 150 for 14.29%, k = 200 for 5.95%. The delta difference between the performances of models obtained from MRMR and KUALA feature sets are (2.00 ± 3.40) % (mean ± std) in terms of F1-measure. The proposed methodology offers ideas for further insights into the best representative molecular descriptors of kinase inhibitors. The results obtained agree with those reported in the literature. Indeed, Abdelbaky et al. highlighted that, in terms of performance of the models trained on kinase inhibitors, the best descriptors (1D and 2D) calculated with PaDEL were the E-states, Autocorrelation, Burden and topological descriptors^20^. The above-mentioned descriptor families have also been found in other works as most representative attributes for kinase inhibitors.

As previously shown in Fig. 4, the descriptors belonging to the “AlogP” class characterize all kinase ligands. This result suggests an important role of hydrophobicity contribution in kinase inhibitor binding process^60–62^. Another class of descriptors shared by kinase dataset analyzed is “Autocorrelation” family. These are topological descriptors related to the spatial distribution of molecular properties and which encode both the molecular structure and the physicochemical properties of a molecule^63,64^. Moreover, “Burden matrix” descriptors together with “Barysz matrix” family account atomic properties, such as electronegativity contributions, and bond orders for pairs of bonded atoms^65–67^. Finally, the “E-state” descriptors provide information about structural attributes, such as the topology and electronic environment of molecular fragments, correlated to various activity responses of the ligands^68^.

From the above discussion, we strongly suggest the use of these descriptor families when exploring the chemical space of the kinase inhibitors for possible target repositioning.

Notably, the computational framework was able to correctly assign kinase inhibitors, currently in clinical trials, to each of known targets in 84% of cases and failed to produce the desired results in the remaining 16%. The comparison between fingerprints with KUALA approach showed that the adoption of ECFP4 allows to obtain better performances for 50% of proteins, MACCS only for 10%, and KRFP for 27%. This is not sufficient to implement an approach entirely based on fingerprint. We are aware that it is possible to improve the performance of the proposed methodology by using alternative features (fingerprints or substructures), nevertheless, the aim of this study is to propose an approach to improve the explainability of ML models. As also reported in the literature, fingerprints present many limitations when used for similarity search or property explorations^69^. The performance of each descriptor or fingerprint should indeed be chosen related to the scientific scope. In our comparison, for example, MACCS showed a very poor prediction, whereas in other cases reported in the literature it outperformed when compared to ECFP4^74^. From the analysis carried out through the use of KRFPs it emerges there is not a net distinction between substructure families of active and inactive compounds (In Supplementary Tables S3, S4 we reported the most frequent mutually exclusive smarts for active and inactive ligands).

Our suggestion when applying such an approach to drug design is to start with selected KRFP “building blocks” and then consider KUALA ML models which rely on molecular descriptors to predict the activity of ligands for a specific kinase.

Furthermore, considering the fundamental aspects for drug repurposing, e.g., high binding site similarity against drug promiscuity, a multi-target priority score (MTPS) has been proposed. This scoring function, accompanied by a repurposing threshold (RT), enabled prioritizing each protein-predicted ligand pair and separating “reliable” from “unreliable” repurposing choice.

Taken together, these results confirmed the high predictive power of the selected ML models in our workflow which offer an approach primarily based on artificial intelligence to be used alone or in combination with other computational techniques to identify drug repurposing candidates.

KUALA was actually designed as a first step tool to guide the rational and explainable molecule selection for drug repurposing and subsequent drug design steps. The concurrent use of KUALA and structure-based methods strengthen the prediction capability. The use of molecular descriptors together with an explainable ML methodology, perfectly fits with the subsequent structure-based evaluation of the binding mode. The combination of binding mode prediction with topological features of ligands is suggested as a powerful instrument to be used in medicinal chemistry.

## Supplementary Information

Supplementary Information 1.

Supplementary Information 2.

## Data Availability

The datasets analysed during the current study are publicly available in the KLIFS repository, https://klifs.net/ and ChEMBL repository, https://www.ebi.ac.uk/chembl. The dataset of all predicted compounds for each kinase are available for download and to browse the results at Shinyapp https://molinfrimed.shinyapps.io/kuala-demo/ and Zenodo, https://doi.org/10.5281/zenodo.6554043. Stand-alone software (demo script and ML models) has been uploaded both on Zenodo (https://doi.org/10.5281/zenodo.7142370) and on GitHub (https://github.com/molinfrimed/multi-kinases).
