# Supplementary material for: KUALA: a machine learning-driven framework for kinase inhibitors repositioning
Source: Sci Rep. 2022 Oct 25;12:17877. doi: 10.1038/s41598-022-22324-8 (PMC9595087; doi:10.1038/s41598-022-22324-8)
Supplement: Supplementary file 1 — Supplementary Information 1. [file 41598_2022_22324_MOESM1_ESM.pdf]

KUALA: A Machine Learning-driven framework for kinase inhibitors repositioning

Giada De Simone, Davide S. Sardina, Maria Rita Gulotta, Ugo Perricone

Supplementary Table S1 – Machine learning methods and related packages in R.

parameter column report the parameters used to take into account class imbalance.

Name	Package	Parameter
NAIVE	naivebayes	prior
LOGISTIC	stats	weights
SVM	e1071	class.weights
C50	C50	weights
RANDOM FOREST	randomForest	classwt
NEURAL NETWORK	neuralnet	weights
XGBOOST	xgboost	scale_pos_weight
KNN	caret	weights
CART	rpart	parms = list(prior)
LASSO	glmnet	weights
RIDGE	glmnet	weights
ELASTICNET	glmnet	weights

Supplementary Table S2 – Docking results

<table>
<tr><th></th><th>Ligand interaction diagram of co-crystallized ligand</th><th>Binding mode of co-crystallized (green) and predicted (orange) ligands</th><th>Ligand interaction diagram of predicted ligand</th></tr>
<tr><td>P31749</td><td>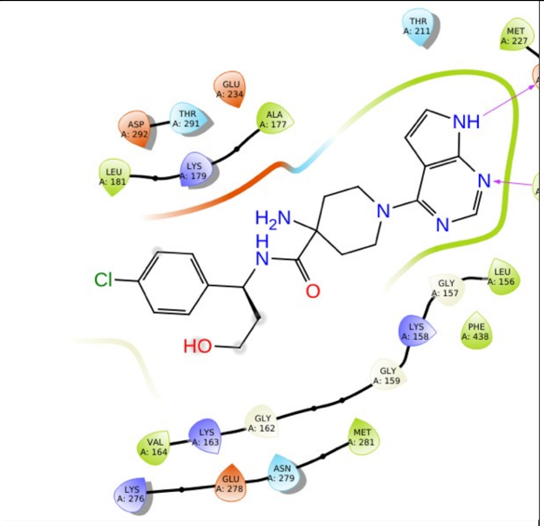
</td><td>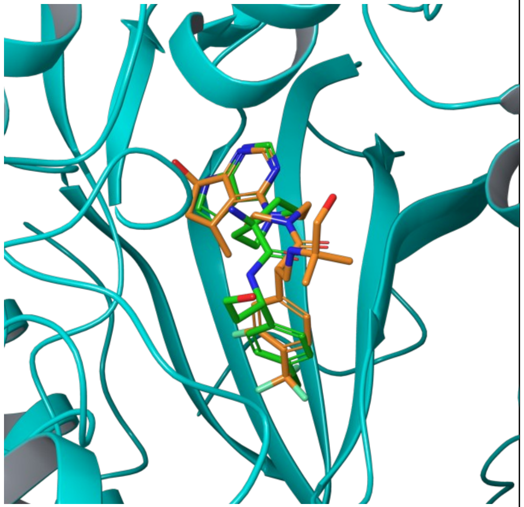</td><td>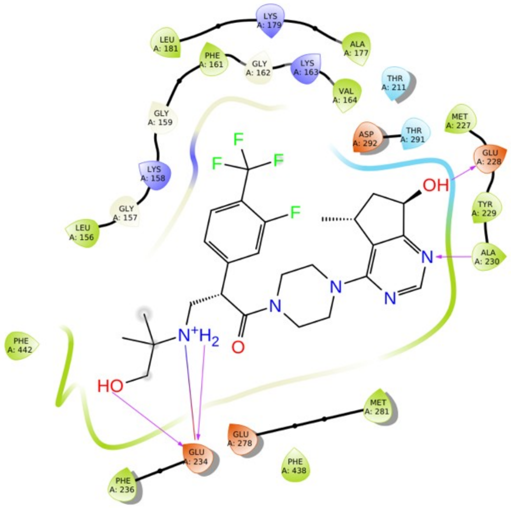
</td></tr>
</table>

P25098

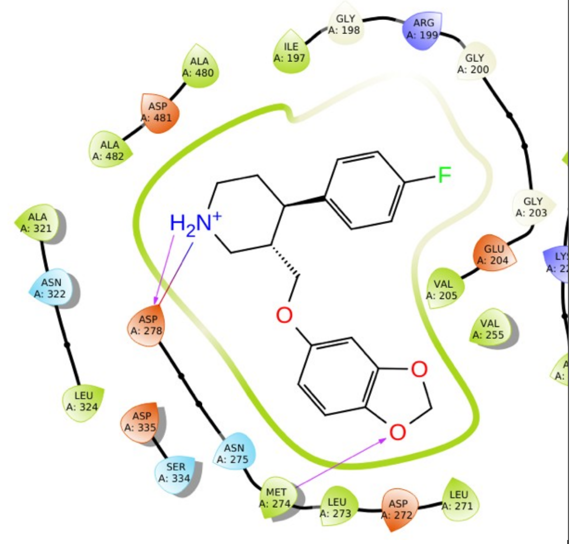

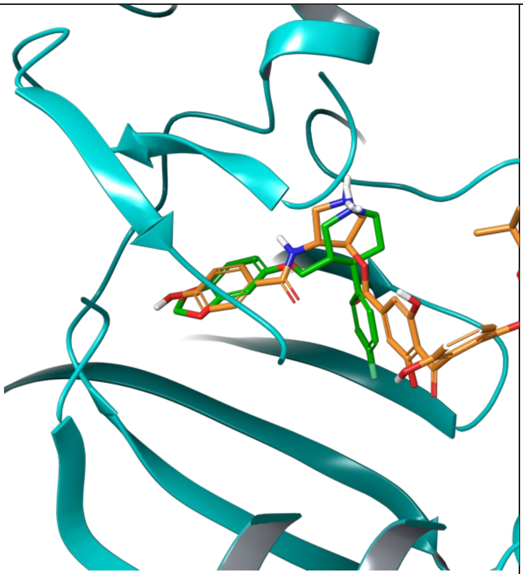

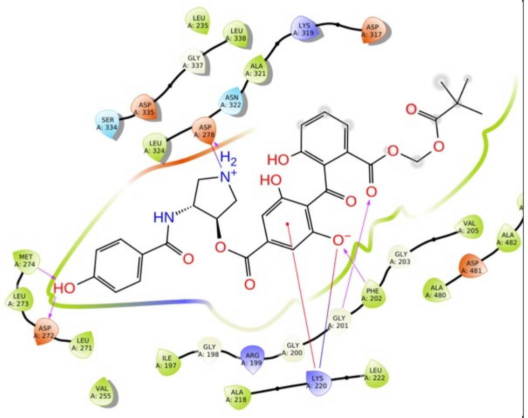

P42336

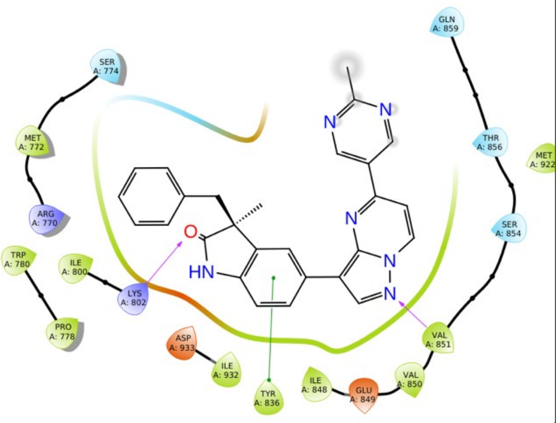

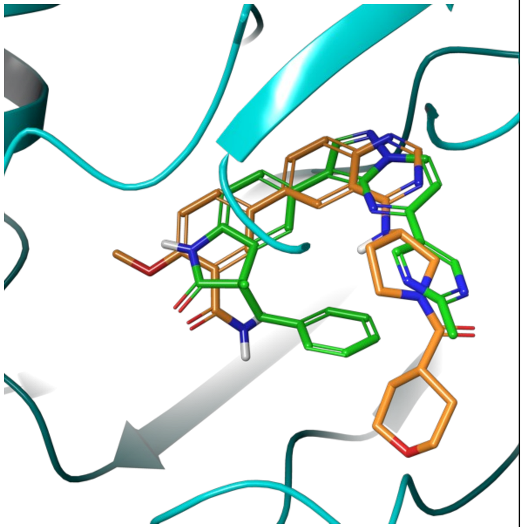

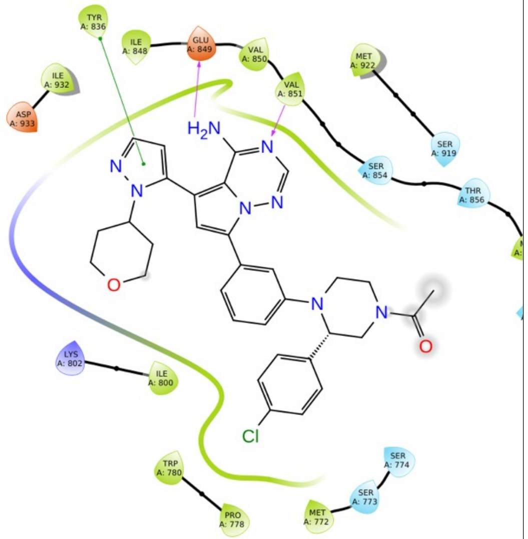

P06493	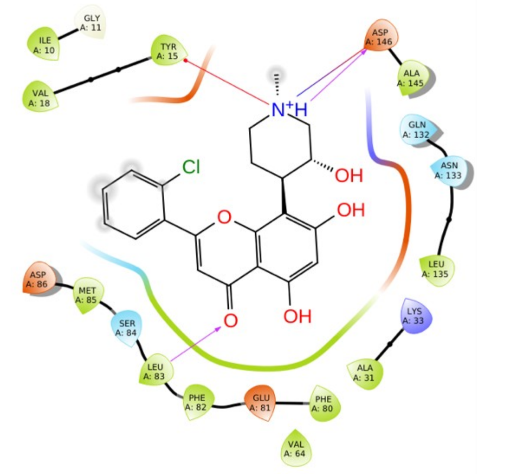	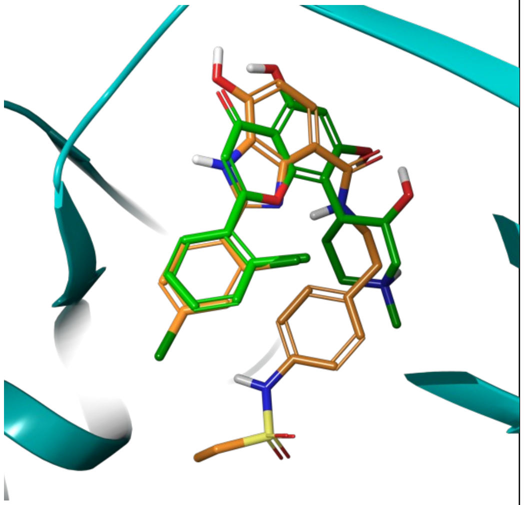	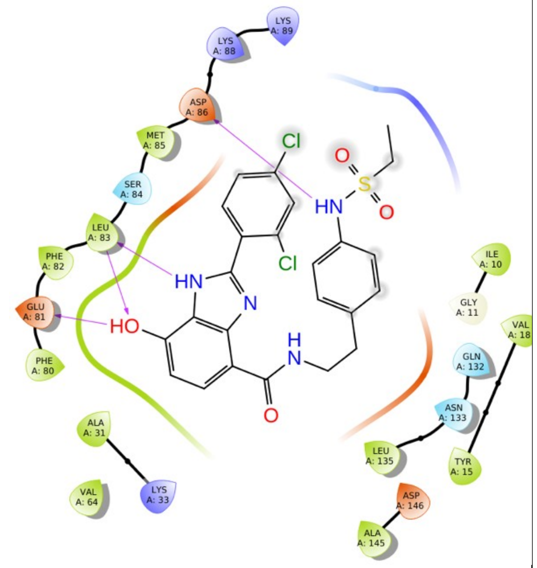
P08069	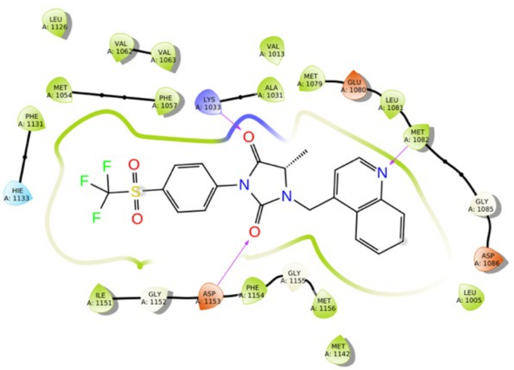	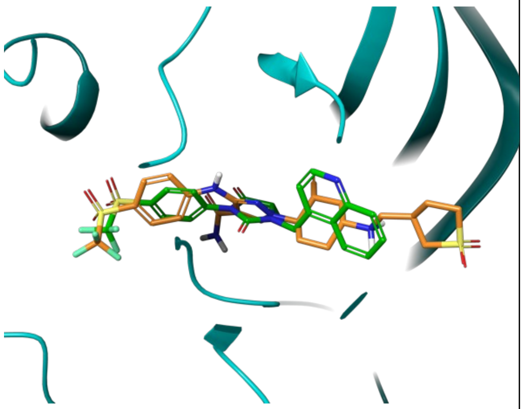	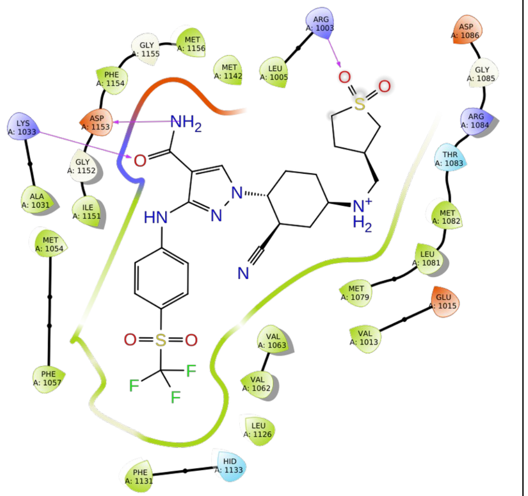

P28482

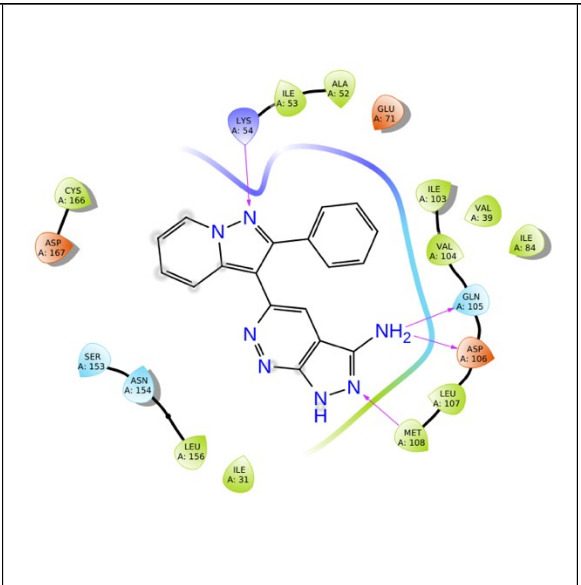

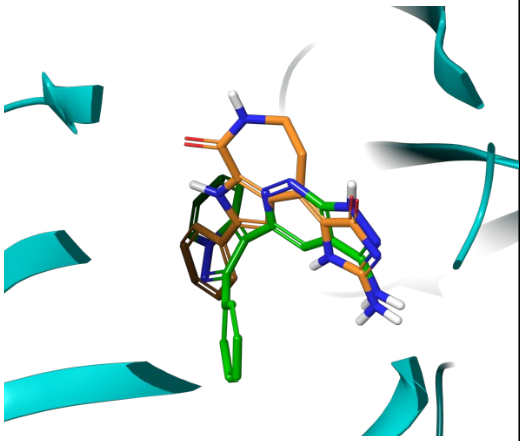

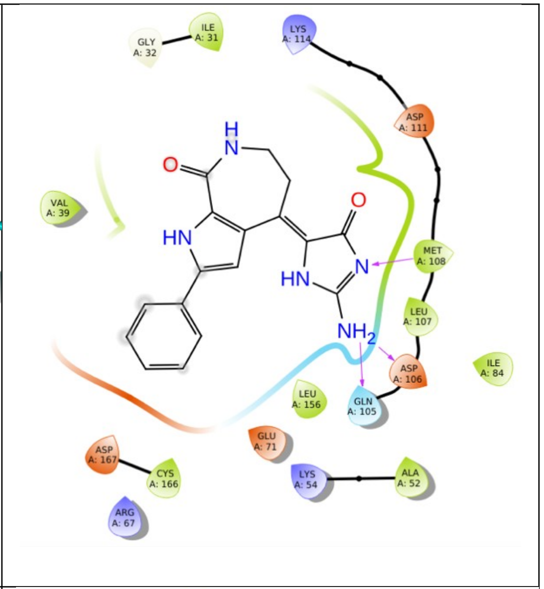

P07333

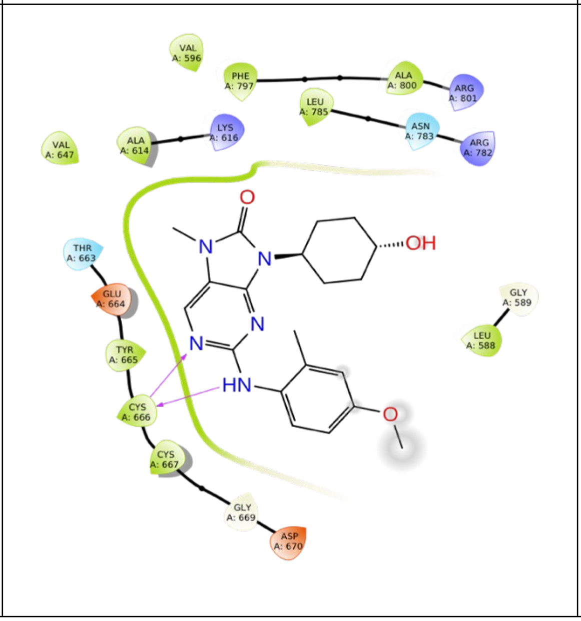

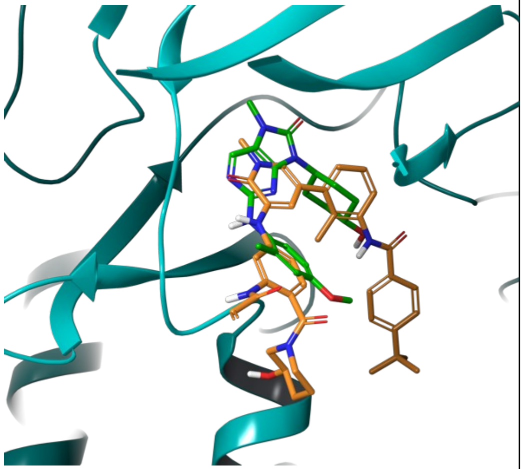

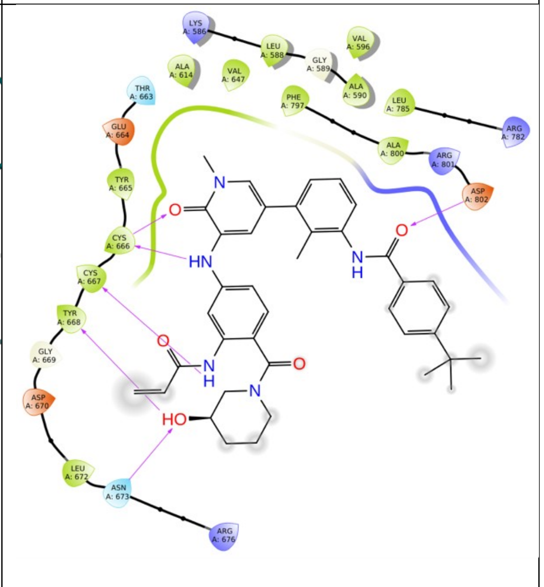

P21802	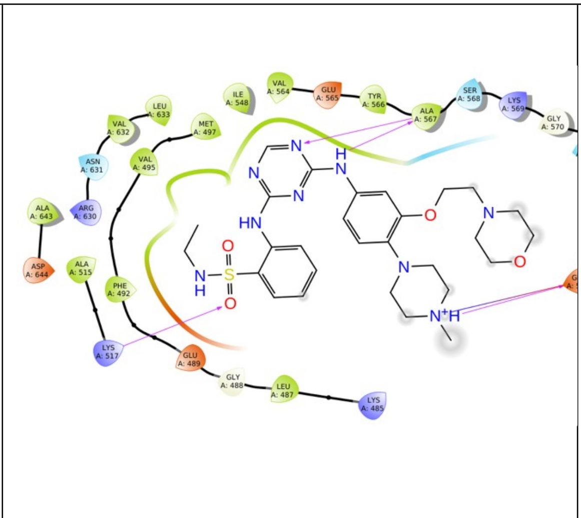	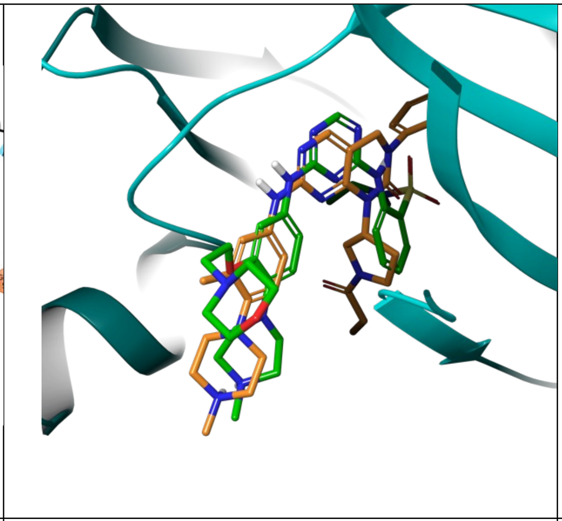	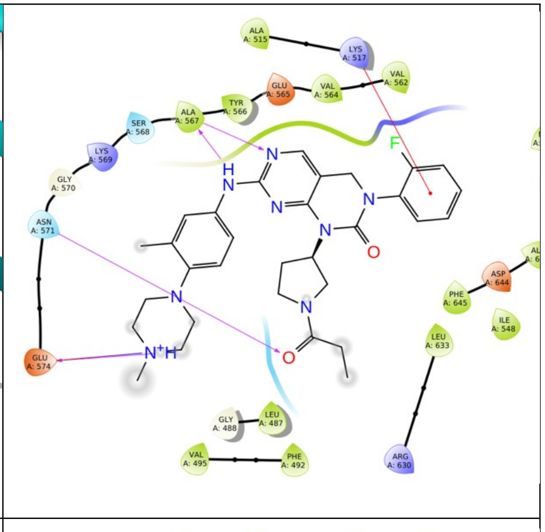
P52333	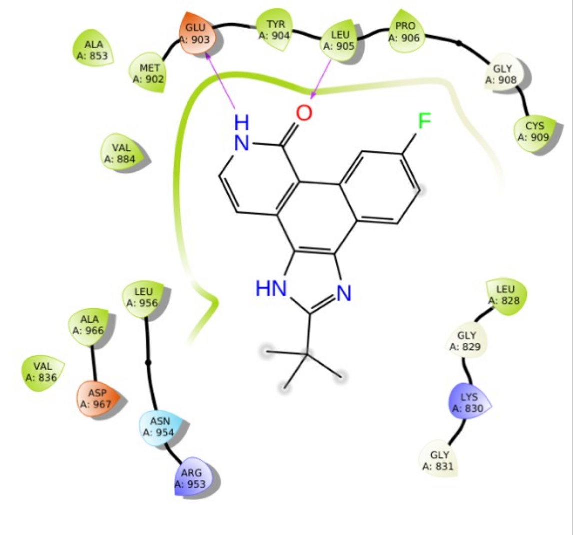	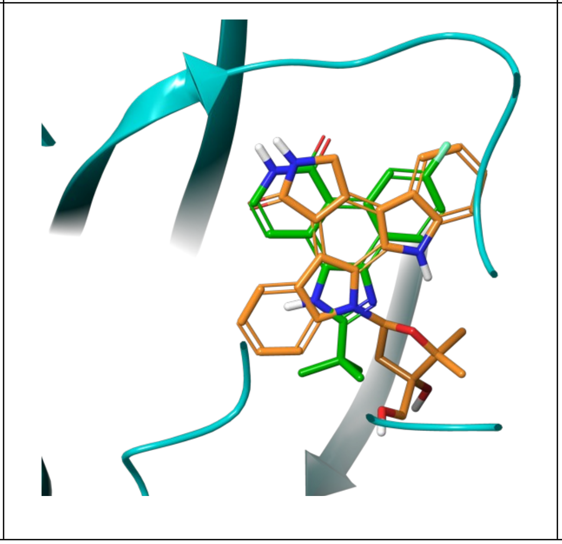	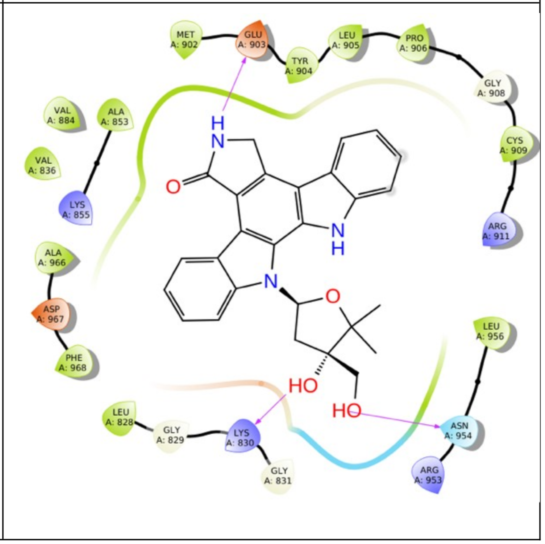

Supplementary Table S3 – Most frequent substructures from Klekota-Roth analysis of active ligands

KR ID	Substructure
KR2395	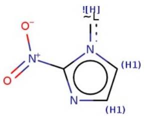
KR4740	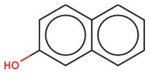
KR2071	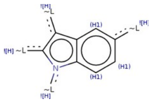
KR554	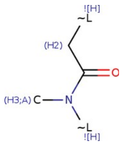
KR3293	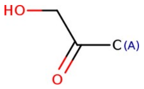
KR1204	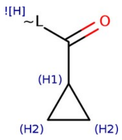
KR4359	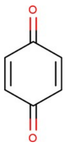
KR790	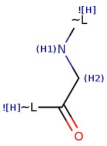

cont.

KR1066	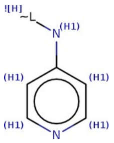
KR4014	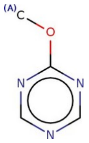
KR4011	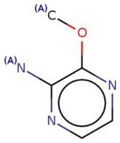
KR3510	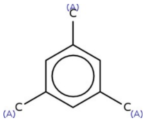
KR3554	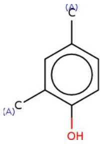
KR4490	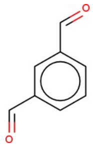
KR776	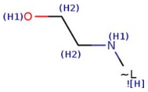
KR4827	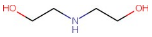
KR1089	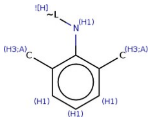

cont.

KR773	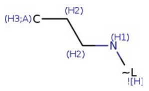
KR3092	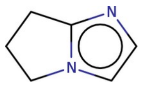
KR4755	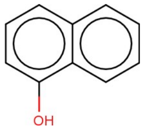
KR136	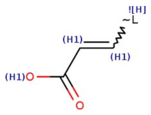
KR1272	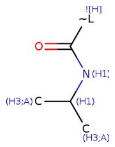
KR1905	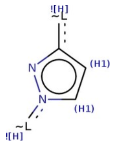
KR3116	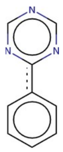
KR3778	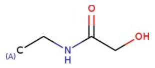
KR3932	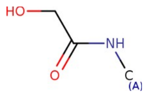

cont.

KR158	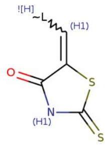
KR1314	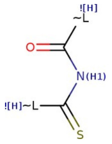
KR3977	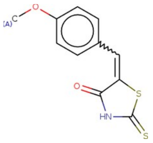
KR1116	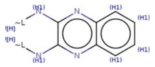
KR4600	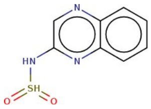
KR915	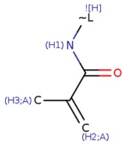
KR4015	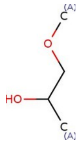
KR3205	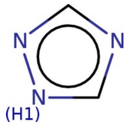
KR2195	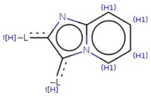

cont.

KR4554	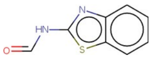
KR993	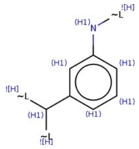
KR3605	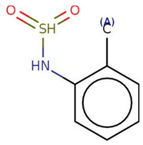
KR3645	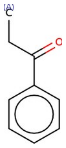
KR4708	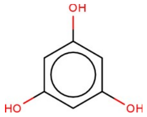
KR1104	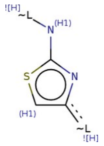
KR2242	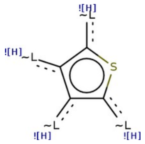
KR2258	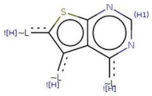

cont.

KR69	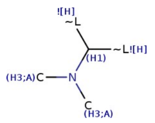
KR374	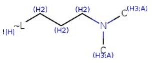
KR37	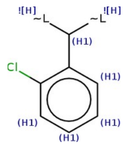
KR564	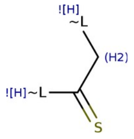
KR3549	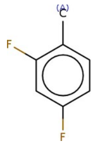
KR775	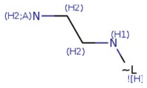
KR4225	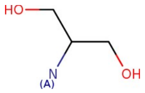
KR3630	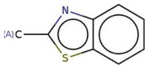
KR2261	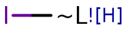

cont.

KR4074	HI
KR4077	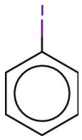
KR4253	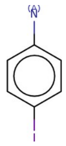
KR4547	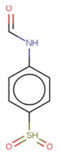
KR908	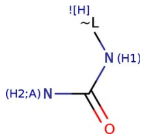
KR2143	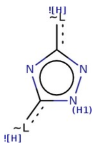
KR3032	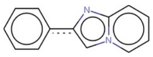
KR2216	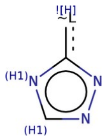

cont.

KR3216	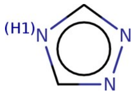
KR1663	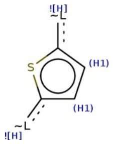
KR4122	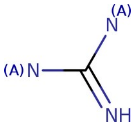
KR842	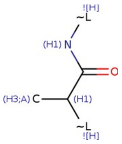
KR3651	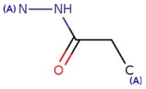
KR3652	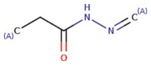

Supplementary Table S4 – Most frequent substructures from Klekota-Roth analysis of inactive ligands

KR ID	Substructure
KR856	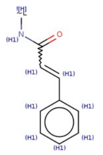
KR2011	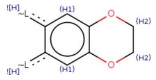
KR4713	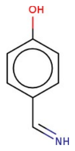
KR590	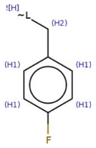
KR3765	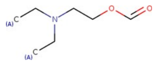
KR4127	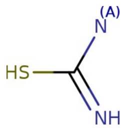
KR1473	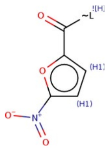

cont.

KR3563	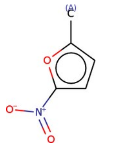
KR354	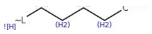
KR3571	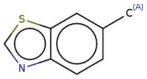
KR1677	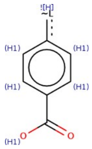
KR1834	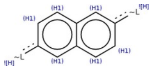
KR3542	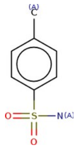
KR4815	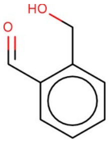
KR2393	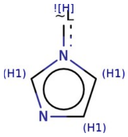

cont.

KR1601	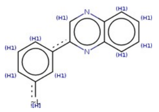
KR2029	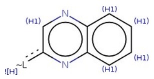
KR3108	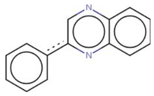
KR1924	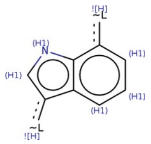
KR1608	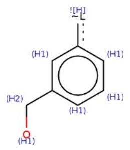
KR837	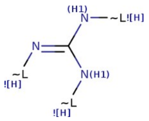
KR4129	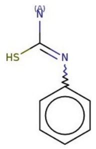
KR2214	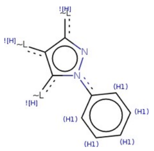

cont.

KR774	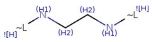
KR3755	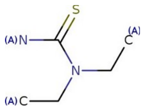
KR3388	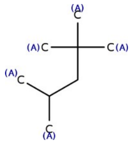
KR1850	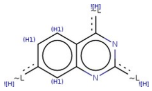
KR1437	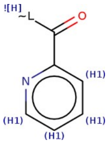
KR3418	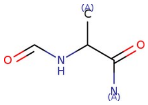
KR3326	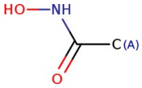
KR2675	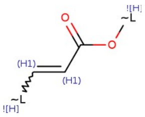

cont.

KR2196	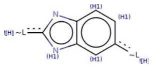
KR459	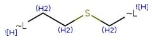
KR594	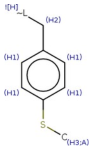
KR351	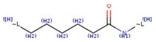
KR518	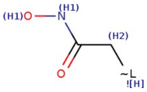
KR3646	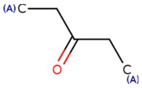
KR1656	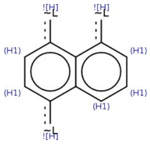
KR2488	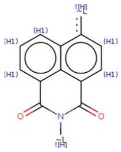

cont.

KR4439	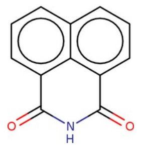
KR4493	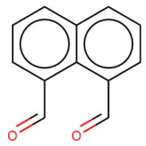
KR189	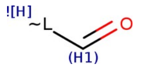
KR585	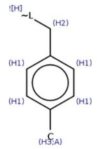
KR802	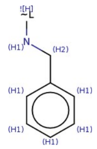
KR803	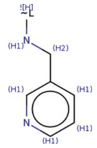
KR3859	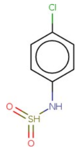
KR1997	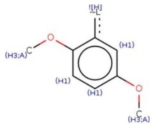
KR1922	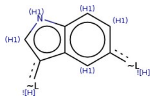
KR3027	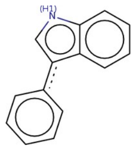

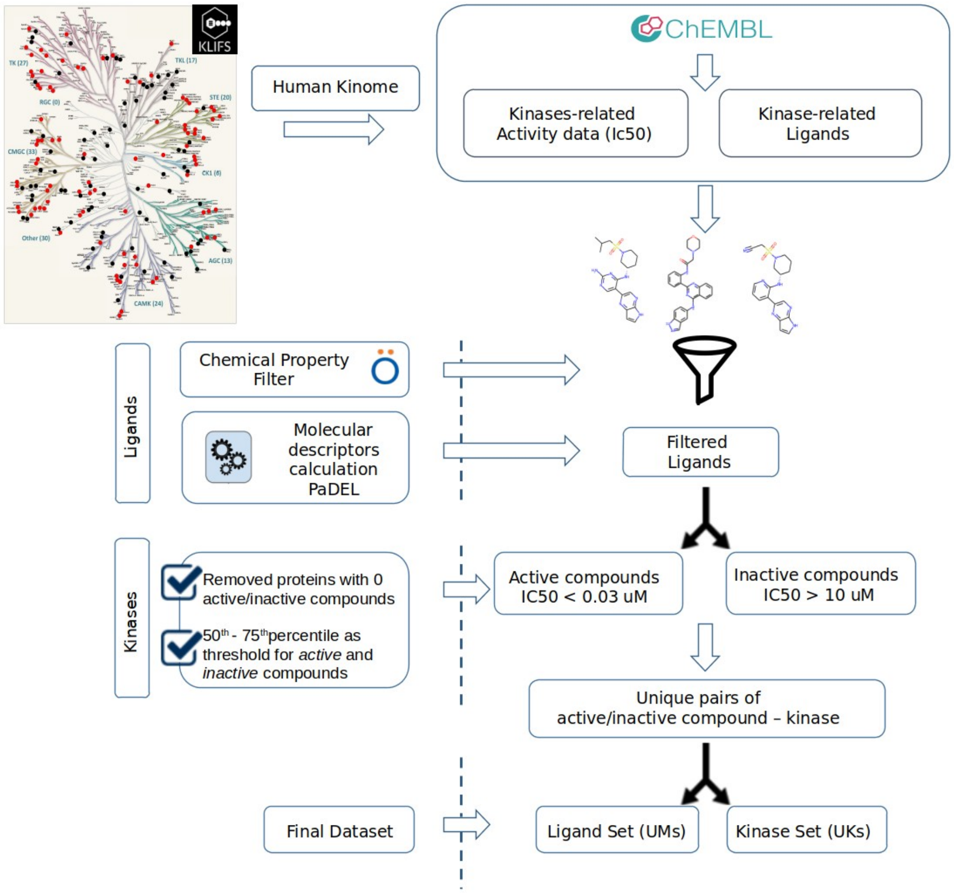

Supplementary Figure S1. Data extraction from KLIFS and ChEMBL databases. Small molecules were filtered by using Schrödinger Suite, then divided into active and inactive compounds through ChEMBL activity data (IC_{50}). Finally, PaD molecular descriptors were computed.

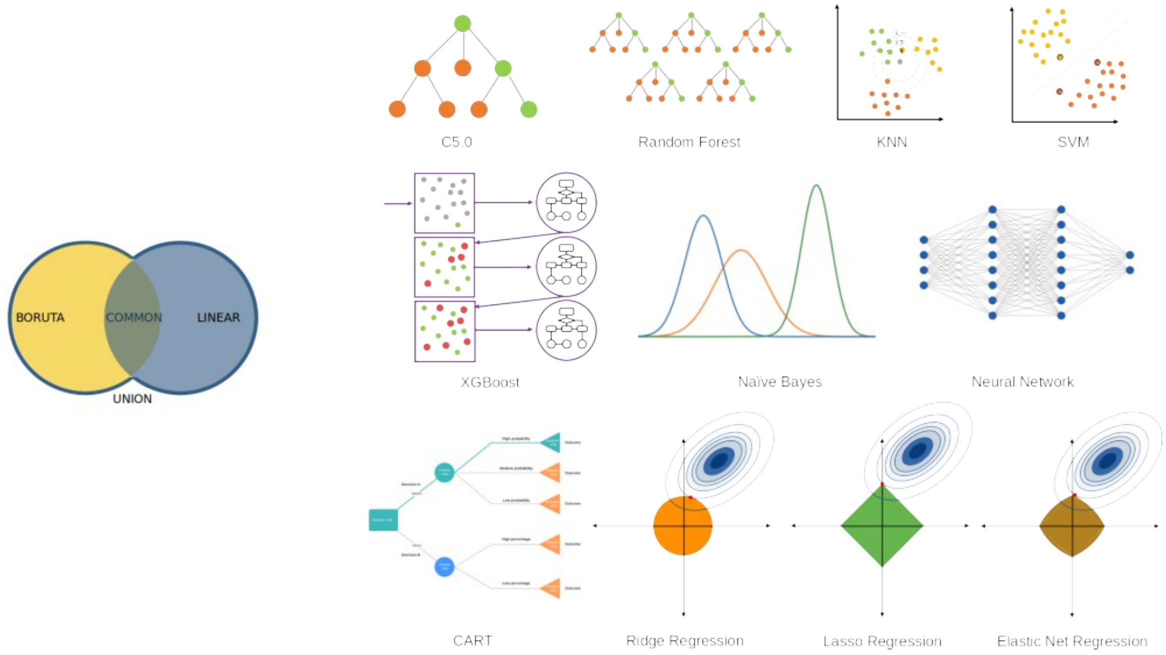

Supplementary Figure S2. (a) Feature set Venn diagram for each kinase. Several feature sets were considered: BAs only, LRs only, intersection between BAs and LRs (Cs), and finally union of BAs and LRs (Us). **(b) ML classification models.** For each protein and feature set, active and inactive ligands were divided into training and test sets. The first ones were used to train the classifiers, while the second ones for the assessment of performance. Hyperparameter tuning was carried out where possible.

$$Ac = \frac{T_P + T_N}{T_P + T_N + F_P + F_N}$$

$$Sp = \frac{T_N}{T_N + F_P}$$

$$Ba = \frac{T_P\left(T_N + F_P\right) + T_N\left(T_p + F_N\right)}{2\left(T_P + F_N\right)\left(T_N + F_P\right)} = \frac{Re + Sp}{2}$$

$$Pr = \frac{T_P}{T_P + F_P}$$

$$Re = \frac{T_p}{T_P + F_N}$$

$$F_1 = \frac{2\times T_P}{2\times T_P + F_P + F_N}$$

Supplementary Figure S3. Performance metrics. Several performance metrics were considered, i.e., Accuracy (Ac), Specificity (Sp), Balanced Accuracy (Ba), Precision (Pr), Recall (Re), F1-measure (F_1) defined as above. T_P, F_P, T_N *and* F_N stand for true positive, false positive, true negative and false negative, respectively.

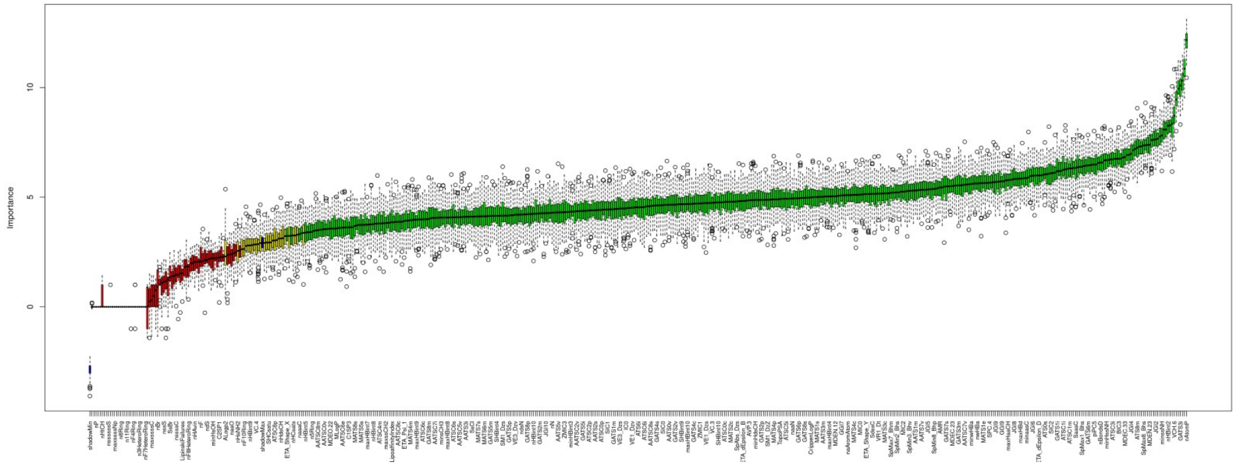

Supplementary Figure S4. Boruta features Importance values distribution for the protein with UniProt ID P35968. The majority of features are confirmed (green), conversely tentative (yellow) or unimportant (red) features are indicated with decreasing Importance values. Minimum and average Importance of shadow variable is shown on the far-left.

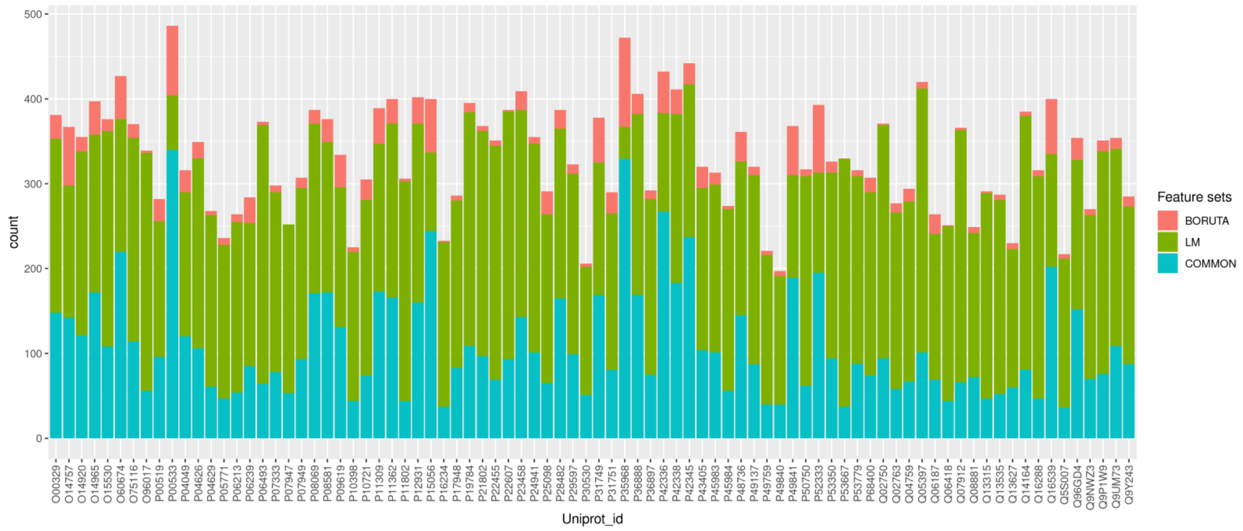

Supplementary Figure S5. Feature computed for protein kinases. Comparison of feature sets used to train the models obtained from Boruta, Linear Regression, and their combinations Union and Common.

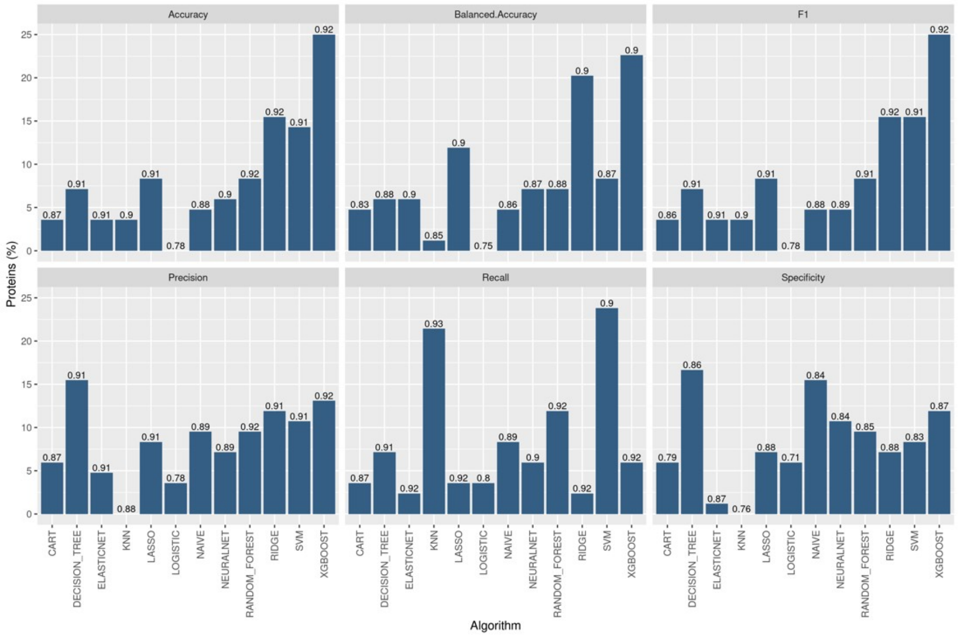

Supplementary Figure S6a – Distribution of best performing algorithms along the 84 UKs for all selected metrics.

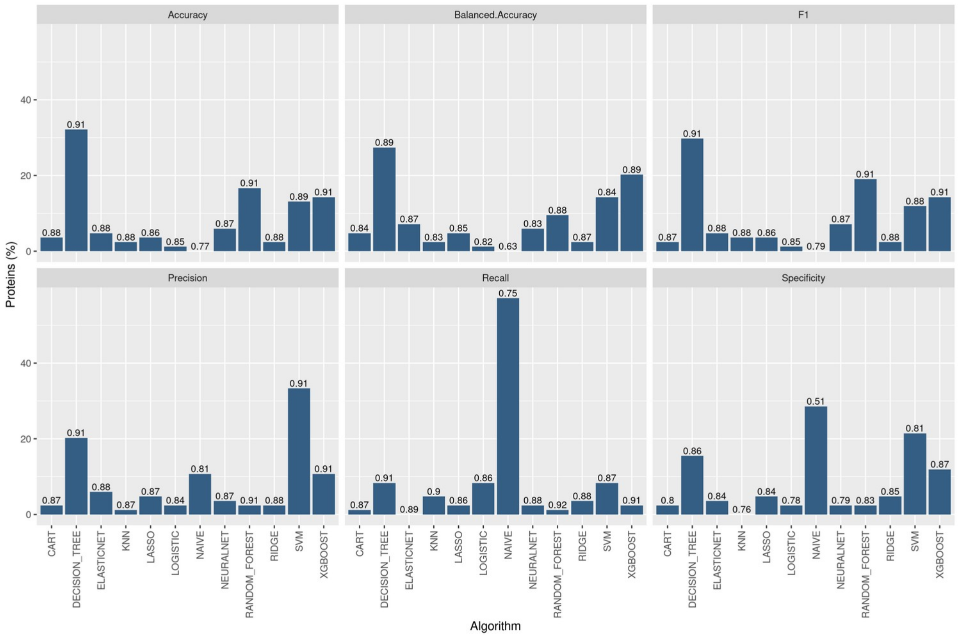

Supplementary Figure S6b – Distribution of best performing algorithms along the 84 UKs for all selected metrics computed with the use of MACCS.

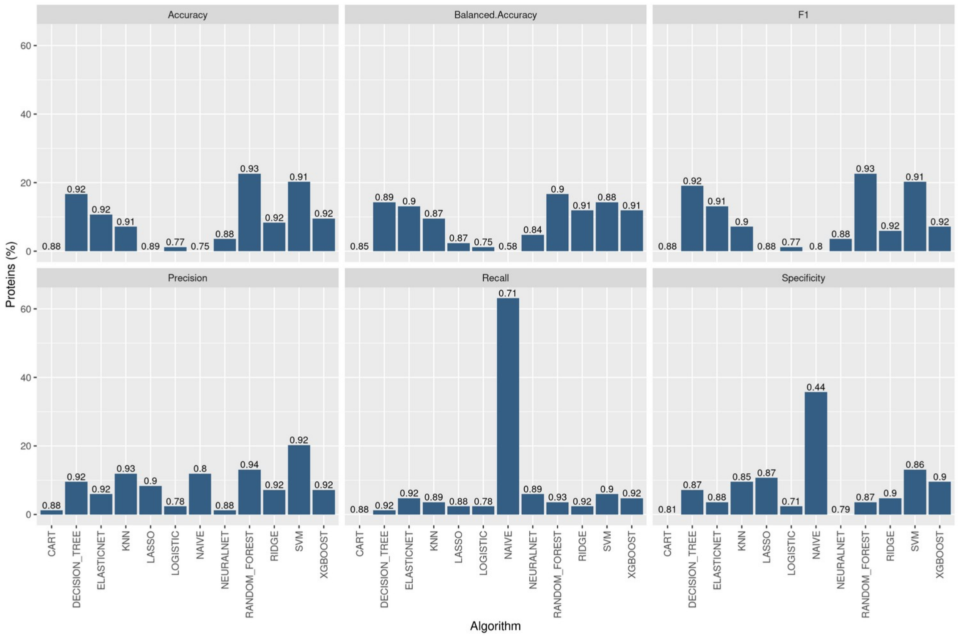

Supplementary Figure S6c – Distribution of best performing algorithms along the 84 UKs for all selected metrics computed with the use of ECFP4.

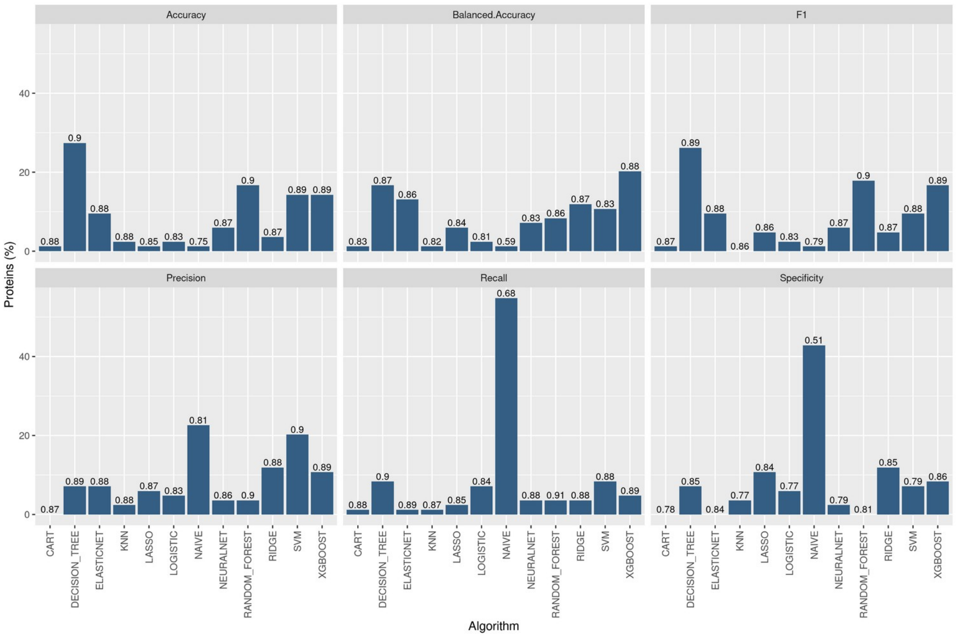

Supplementary Figure S6d – Distribution of best performing algorithms along the 84 UKs for all selected metrics computed with the use of KRFP.

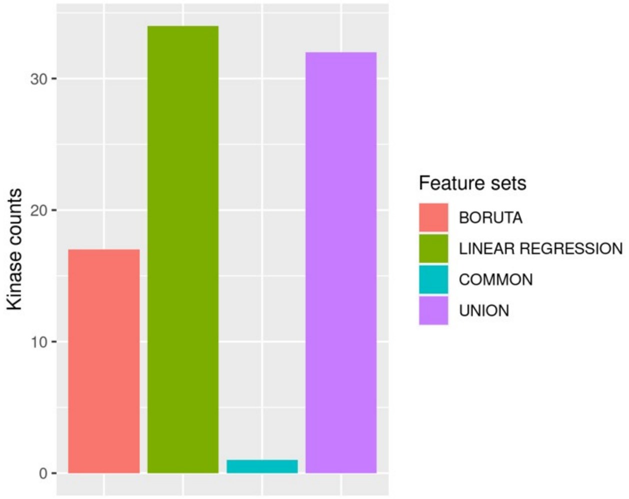

Supplementary Figure S7. Best feature set distribution along 84 UKs.

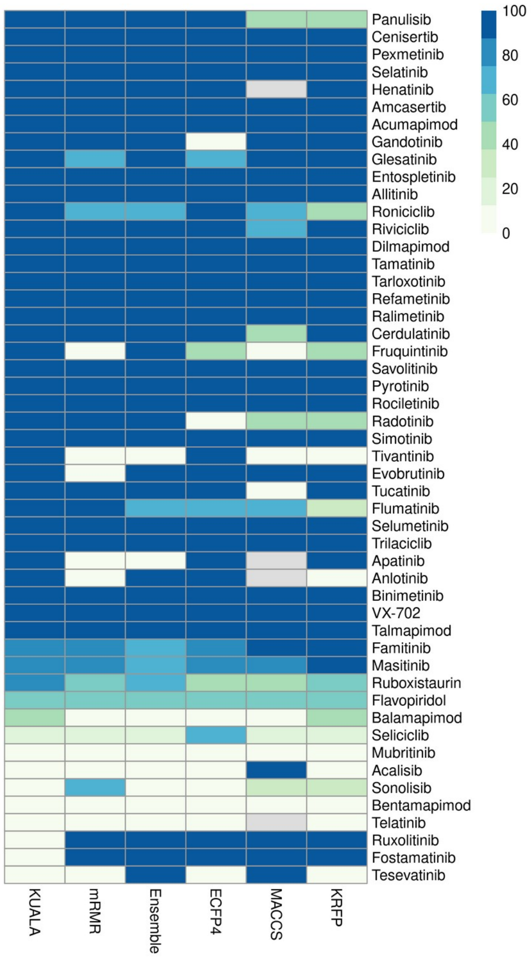

Supplementary Figure S8 – PKIDB and MedChem Express datasets of kinase inhibitors were fed to the 84 models obtained from mRMR, Ensemble, ECFP4, MACCS, and KRFP in order to compare their classification results with KUALA framework. The colorbar in the heatmap represents the percentage of match between the predicted and the validated kinase inhibitors, for each method. MACCS didn’t provide any prediction for 4 drugs (Henatinib, Apatinib, Anlotinib, Telatinib) since the models related to the corresponding kinases were not evaluable (colored in grey).

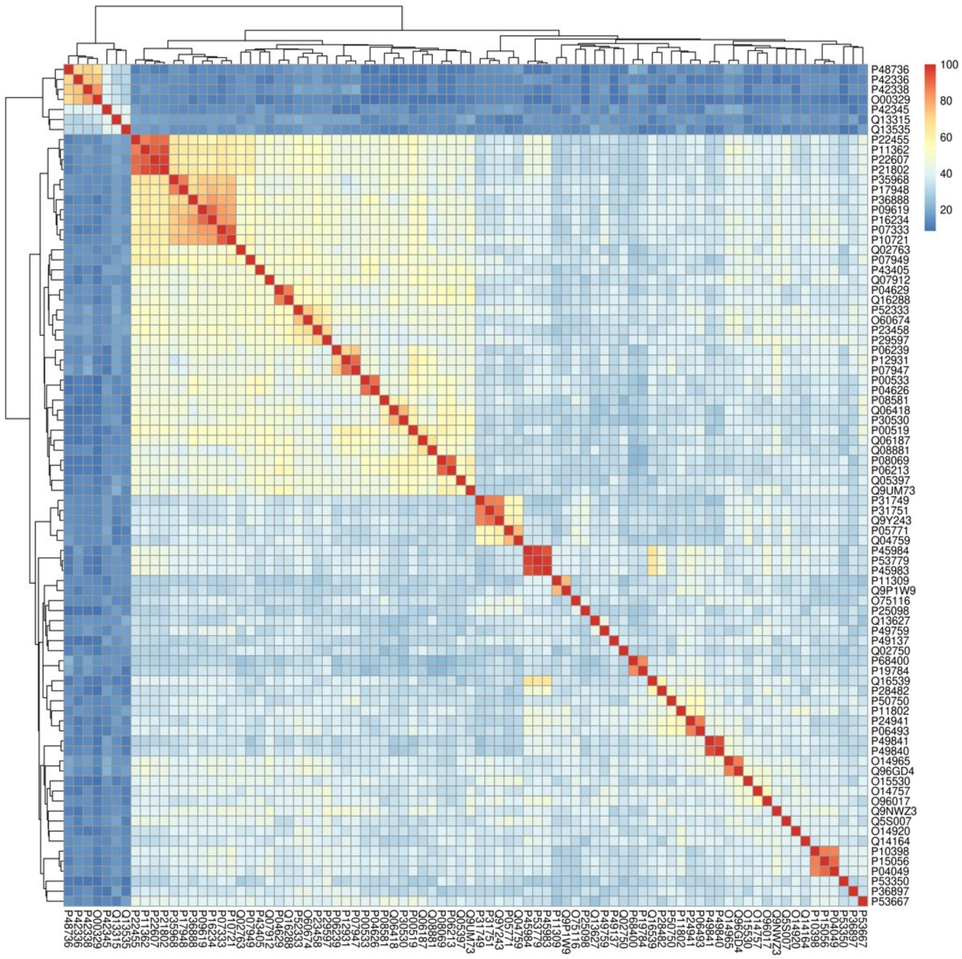

Supplementary Figure S9 – Heatmap showing binding pocket pairwise *identity* considering all 84 kinases based on binding pocket sequence *identity*. The colorbar reports similarity range from 0 (blue) to 100 (red).

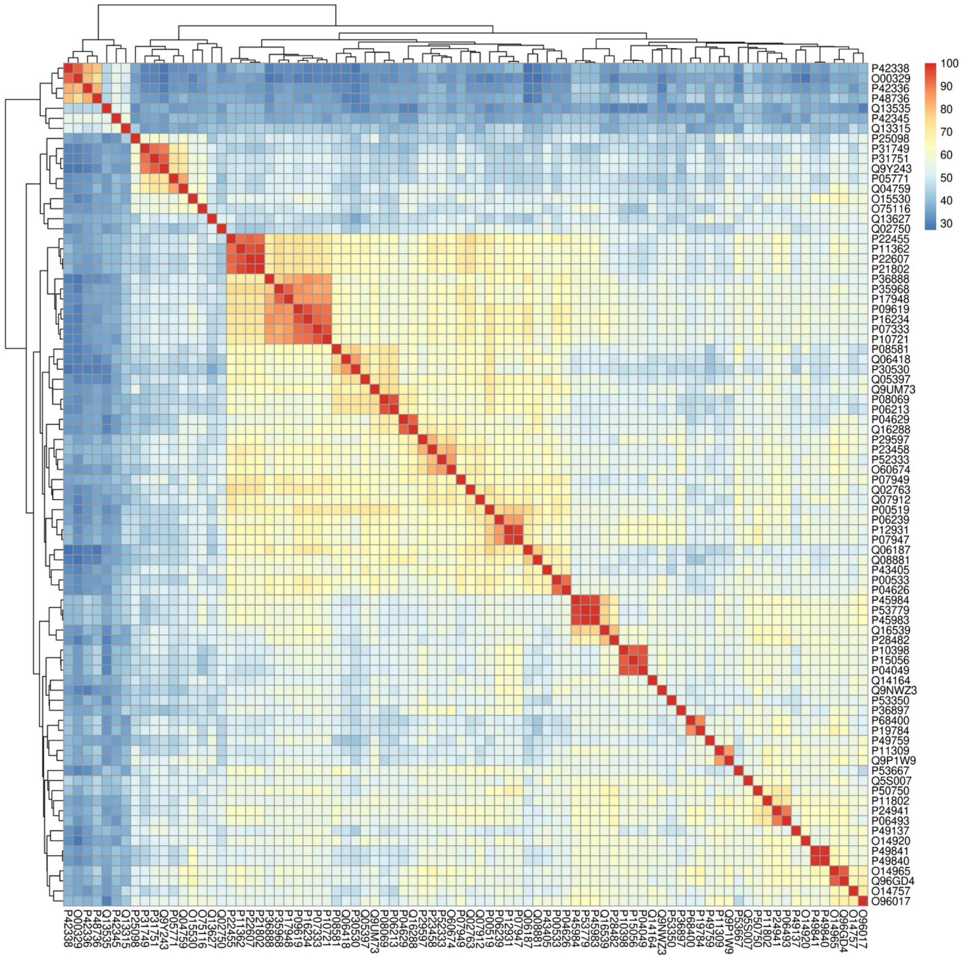

Supplementary Figure S10. Heatmap showing binding pocket pairwise *similarity* considering all 84 kinases based on binding pocket sequence *similarity*. The colorbar reports similarity range from 0 (blue) to 100 (red).

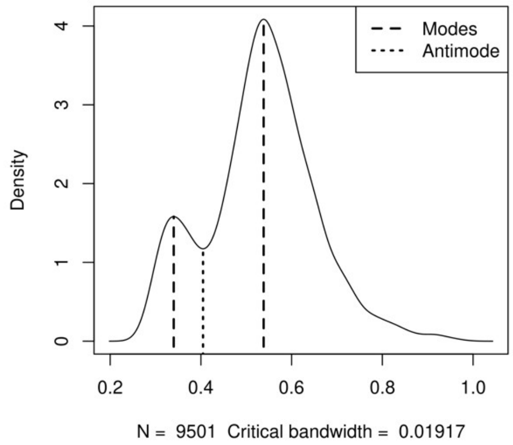

Supplementary Figure S11. Overall density distribution plot of multi-target priority scores (MTPS). Modes and Antimodes are indicated by dashed and dotted lines, respectively. On the X-axis are reported MTPS values while on the Y-axis their distribution.

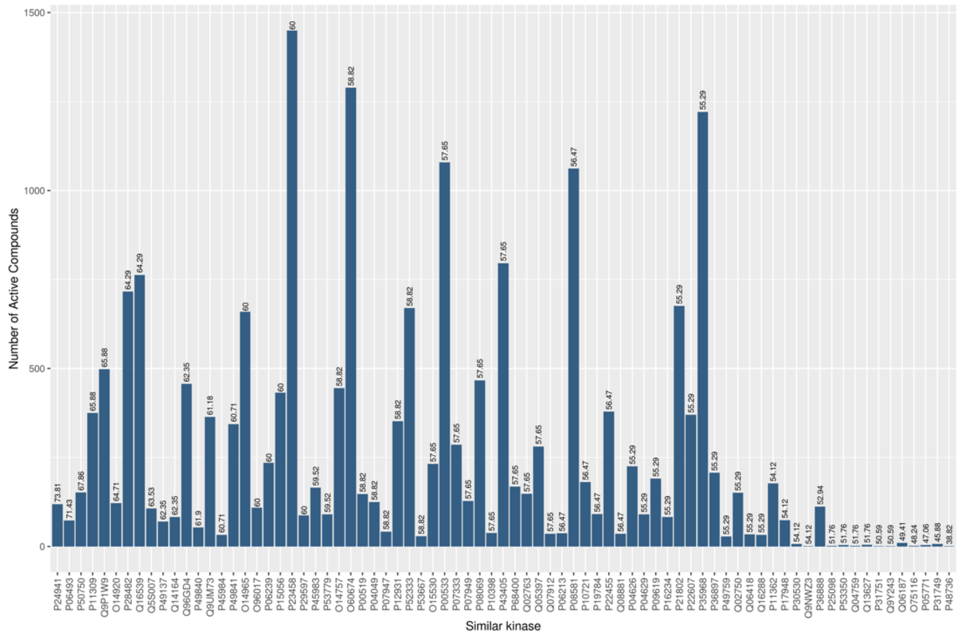

Supplementary Figure S12 – Actives distribution as a function of the most CDK4-similar proteins. The high number of predictions depends on cardinality of CDK4-similar kinase set.

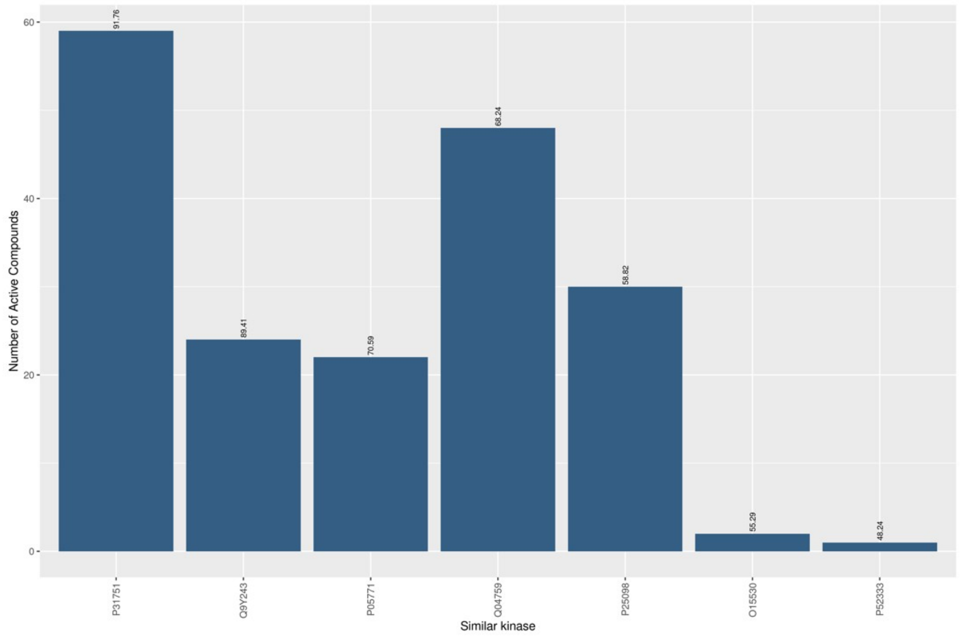

Supplementary Figure S13 – Actives distribution as a function of the most AKT1-similar proteins. The low number of potential repurposable ligands depends on the small cardinality of similar kinase set.
